# Molecular basis of Tick Born encephalitis virus NS5 mediated subversion of apico-basal cell polarity signalling

**DOI:** 10.1042/BCJ20220037

**Published:** 2022-06-22

**Authors:** Airah Javorsky, Patrick O. Humbert, Marc Kvansakul

**Affiliations:** 1 Department of Biochemistry & Chemistry, La Trobe Institute for Molecular Science, La Trobe University, Melbourne, Victoria 3086, Australia; 2 Research Centre for Molecular Cancer Prevention, La Trobe University, Melbourne, Victoria 3086, Australia; 3 Department of Biochemistry & Pharmacology, University of Melbourne, Melbourne, Victoria 3010, Australia; 4 Department of Clinical Pathology, University of Melbourne, Melbourne, Victoria 3010, Australia

**Keywords:** cell polarity, crystallography, isothermal titration calorimetry, PDZ domain, virology

## Abstract

The Scribble (Scrib) protein is a conserved cell polarity regulator with anti-tumorigenic properties. Viruses like the Tick-born encephalitis virus (TBEV) target Scribble to establish a cellular environment supporting viral replication, which is ultimately associated with poor prognosis upon infection. The TBEV NS5 protein has been reported to harbour both an internal as well as a C-terminal PDZ binding motif (PBM), however only the internal PBM was shown to be an interactor with Scribble, with the interaction being mediated via the Scribble PDZ4 domain to antagonize host interferon responses. We examined the NS5 PBM motif interactions with all Scribble PDZ domains using isothermal titration calorimetry, which revealed that the proposed internal PBM did not interact with any Scribble PDZ domains. Instead, the C-terminal PBM of NS5 interacted with Scrib PDZ3. We then established the structural basis of these interactions by determining crystal structures of Scrib PDZ3 bound to the NS5 C-terminal PBM. Our findings provide a structural basis for Scribble PDZ domain and TBEV NS5 interactions and provide a platform to dissect the pathogenesis of TBEV and the role of cell polarity signalling using structure guided approaches.

## Introduction

Cell polarity is the fundamental process that dictates the shape and orientation of a cell via the asymmetric distribution of cellular constituents such as proteins, lipids and carbohydrates [1–3]. The correct distribution of biomacromolecules leads to the establishment of apical-basal cell polarity in epithelial cells, and affects a range of important cellular processes and signalling pathways including apoptosis, vesicle trafficking, interferon (IFN) responses, cell proliferation, JAK-STAT signalling and migration [2, 4]. Notably, the loss of cell polarity has tumorigenic effects and is also associated with poor outcomes during viral infection [2, 5]. Epithelial apical-basal polarity is controlled by the antagonistic interactions between three multi-protein modules, the Par, Crumbs and Scribble modules. The Scribble module is mainly composed of Scribble (Scrib), Drosophila disc large tumour suppressor (Dlg) and Lethal giant larvae (Lgl) proteins, which are localised at the epithelial tight junction of the apical and basolateral region of the cell [2, 6].

Scribble is part of the LAP (leucine-rich repeats and PDZ domain) protein family, with sixteen LLRs (Leucine-rich repeats), two LAP-specific domains (LAPSDa and LAPSDb) and four PDZ domains ((Post-synaptic density protein (PSD95), Drosophila disc large tumour suppressor (Dlg), and zonula occludens-1 protein (ZO-1)).

PDZ domains are ∼90 amino acids long and consist of 5–6 β-strands and 2–3 α-helices with a conserved fold comprising a canonical ligand-binding groove formed by the α2 helix and the β2, β4 and β5 strands [7]. In particular, the Scrib PDZ domains are mediators for the vast majority of host and pathogenic ligand interactions via short sequences termed PDZ binding motifs (PBMs), thus influencing the localisation of the Scribble module within the cytoplasm [2, 6]. PBM sequences are divided into three major classes based on their ligand recognition sequences, as follows: Class I has an X-T/S-X-V/L_COOH_ motif, Class II with an X-ψ-X-ψ_COOH_ motif, and Class III interacting with X-D/E-X-ψ_COOH_ motif [8], in which X is an unspecified amino acid and ψ refers to any hydrophobic amino acid.

The zoonotic Tick-born encephalitis virus TBEV encodes for the NS5 protein, which has been shown to interact with Scribble in a PDZ domain dependent manner [9, 10]. Mainly transmitted to mammals by insect vectors, TBEV causes severe encephalitis in humans, with a mortality rate of 20–30%, which is high among the flavivirus family [11, 12]. It is a single-stranded RNA virus that encodes a single polyprotein, which undergoes post-translational modification to yield three structural and seven non-structural (NS) proteins in the order C-prM-E–NS1–NS2A–NS2B–NS3–NS4A–NS4B–NS5 [12, 13]. TBEV NS5 is a 903 amino acid protein (103 kDa), which makes it the largest and most conserved of the *flaviviridae* proteins [14]. It contains a methyltransferase (MTase) domain at the N-terminus and a RNA-dependent RNA polymerase (RdRp) domain at the C-terminus that are required for capping and synthesis of the viral RNA genome [15].

Although NS5 features two recognisable PBMs, only the internal PBM located within the MTase domain was shown by a yeast-two hybrid screen to bind to Scribble via the PDZ4 domain, whereas the conventional C-terminal PBM did not interact with Scribble [9, 10]. Internal PBMs are unusual, however their existence highlights the plasticity inherent in PDZ domains that can be exploited for both recognition and regulation [16–18]. NS5 was shown to compete with β-PIX for Scribble, thus inhibiting neurite growth in PC12 cells [19], and providing a potential mechanism for TBEV mediated subversion of host cell polarity signalling. TBEV NS5 is not the only flaviviral NS5 protein shown to interact with PDZ-containing proteins, with the closely related West Nile Virus (WNV) and Dengue virus (DENV) NS5 also binding to Scrib, ZO-1 and RIMS2 [20]. These data suggested a role for apical-basal cell polarity during host anti-viral immune responses and in viral pathogenesis.

Here we provide a structural basis for the subversion of the apical-basal polarity Scribble by the TBEV NS5 and demonstrate biochemically and through structural studies a new interaction of the TBEV NS5 C-terminal PBD with the PDZ3 domain of Scribble.

## Results

### TBEV NS5 C-terminal PBM interacts with SCRIB PDZ3

**Figure 1.**
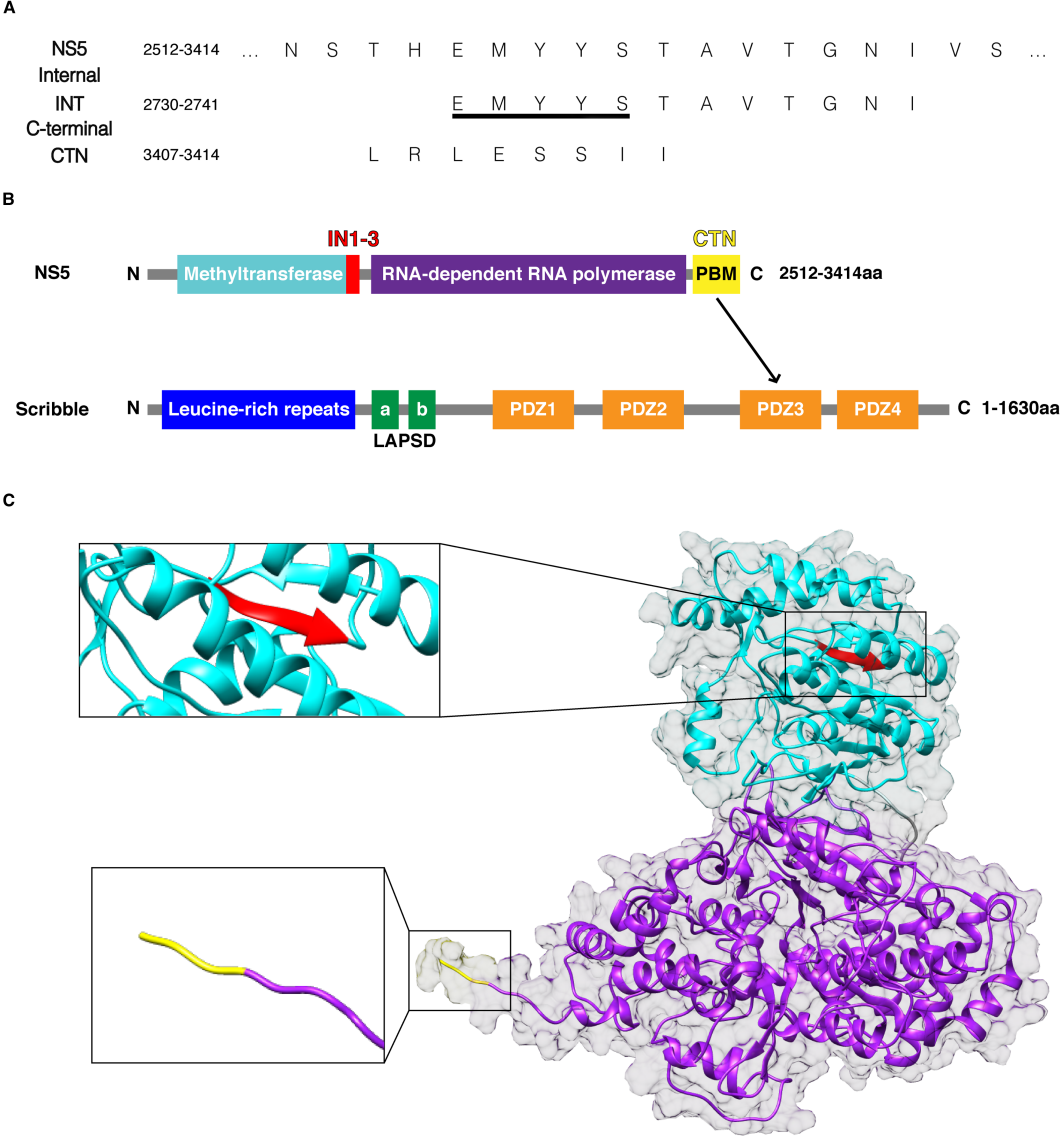
Domain organization of TBEV NS5 and human Scribble. (**A**) PBM sequences of TBEV NS5. Full length NS5 comprises residues 2512–3414 based on the TBEV polyprotein (Uniprot accession number Q01299). INT (underlined) was previously reported to be the internal binder [10], and a sequence covering INT with a C-terminal flanking sequence was selected for further investigation. Our findings support the C-terminal binding, not the internal. (**B**) Schematic outline of TBEV NS5 and Scribble interaction. Red highlights the internal PBM that did not appear to bind. (**C**) Alphafold structure of full length TBEV NS5 (Apache License, Version 2.0). Methyltransferase in cyan, RNA-dependent RNA polymerase in purple, suggested internal in PBM red and C-terminal PBM in yellow.

**Figure 2.**
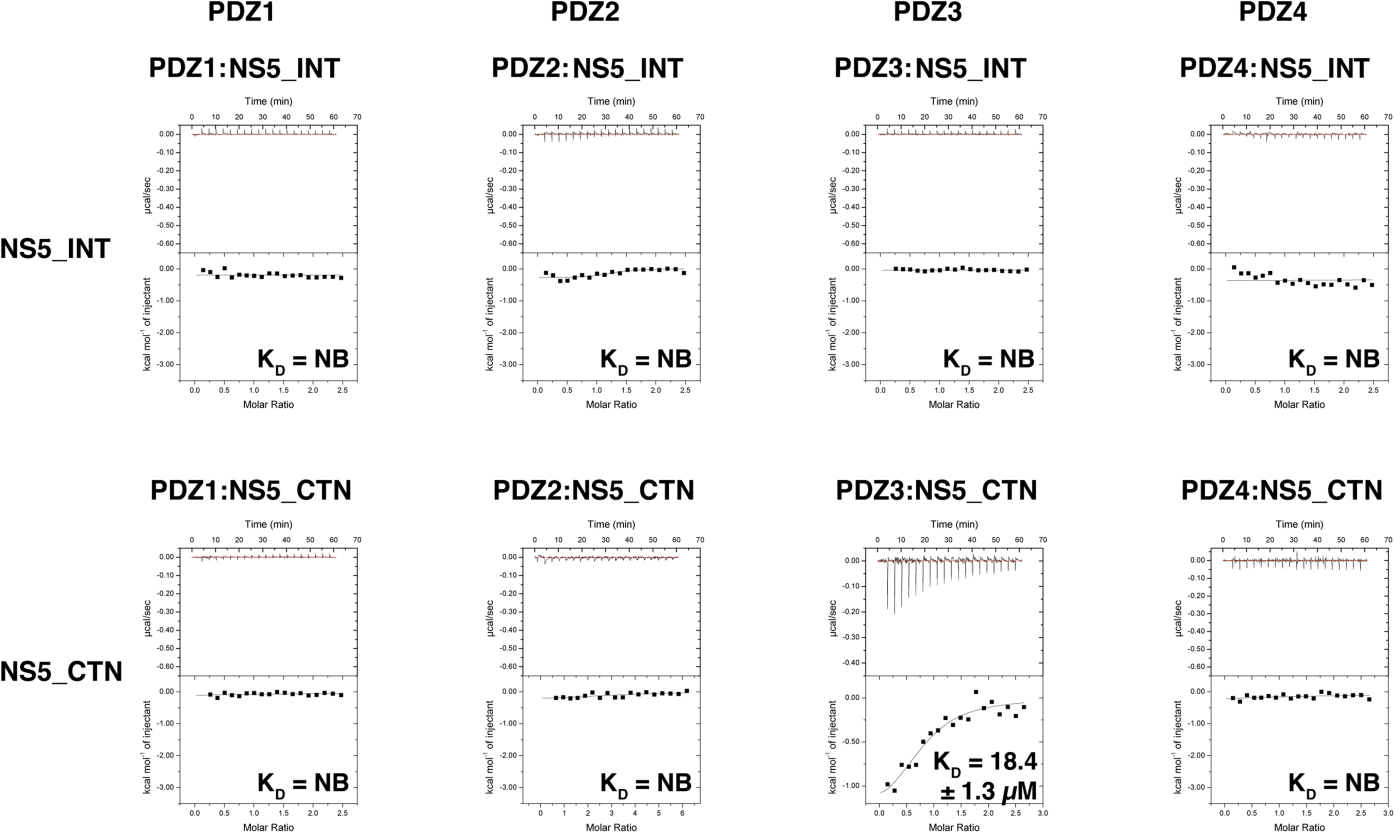
Interactions of Scribble PDZ domains with NS5 PBM peptides. Affinities were measured using isothermal titration calorimetry (ITC) and the raw thermograms are shown. *K*_D_ values (in µM) are the means of three experiments ± SD.

To understand the molecular and structural basis of the human Scribble:NS5 interaction, we systematically examined the ability of recombinant human Scribble PDZ domains to bind to peptides encoding for identified PBM motifs in TBEV NS5 (Figure 1). For this purpose, we used synthethic peptides spanning two putative regions identified as interactors for Scrib PDZ domains: a canonical C-terminal PBM termed NS5_CTN, and a sequence spanning a previously identified internal PBM including flanking residues on both sides of the core binding motif (termed NS5_INT) to account for the fact that as an internal PBM sequence it would be expected to interact in the context of a longer peptide sequence with no involvement of a C-terminal carboxylate group. Isothermal titration calorimetry revealed that only the C-terminal PBM NS5_CTN bound to SCRIB PDZ3 with a *K*_D_ value of 18.4 ± 1.3 µM. Examination of the thermodynamic binding parameters of NS5_CTN peptides binding to the SCRIB PDZ domains indicated that the SCRIB PDZ domain:PBM interactions were entropy-driven by a more favourable −TΔS, (Table 1, Supplementary Figure S1). In contrast, no interaction was observed for NS5_INT. (Table 1, Figure 2, Supplementary Figure S4). With no evidence for binding observed for the internal PBM sequence, we surmised that this motif is likely not to be active in the context of the full protein [10] (Table 1, Figure 2). Examination of the predicted Alphafold full length TBEV NS5 protein structure revealed that this sequence (NS5_INT) is located in the middle of a β-sheet, preventing the exposure of the PBM residues for canonical binding to SCRIB PDZ domains (Figure 1C). The predicted Alphafold TBEV NS5 structure superimposed with DENV NS5 (PDB: 4V0Q) with an RMSD of 0.87 Å over the Cα backbone atoms. DENV NS5 featured the highest sequence similarity (58%) between other *flaviviridae* NS5 protein structures including; ZIKA (PDB: 5U0B), JEV (PDB: 4K6M) and DENV. For yellow fever, DENV, JEV, ZIKA and WNV NS5, these related NS5 sequences displayed 62–87% sequence identity in the region spanning the putative NS5_INT motif, suggesting that in all cases this motif would be located within an existing β-sheet. We note that the presence of this motif in a β-sheet does not preclude the possibility of exchange with a different conformational state that would be amenable to an interaction with a PDZ domain.

**Table 1:** Interactions of wild type and mutant SCRIB PDZ domains with TBEV NS5 PBM peptides

SCRIB domains with ligands	N	*K*_D_ (µM)	ΔG (kcal mol^−1^)	ΔH (kcal mol^−1^)	−TΔS (kcal mol^−1^)
PDZ1:CTN	NB	NB	NB	NB	NB
PDZ2:CTN	NB	NB	NB	NB	NB
PDZ3:CTN	1.0 ± 0.2	18.4 ± 1.3	−6.4 ± 0.01	−1.1 ± 0.4	5.3 ± 0.4
PDZ4:CTN	NB	NB	NB	NB	NB
PDZ1:INT	NB	NB	NB	NB	NB
PDZ2:INT	NB	NB	NB	NB	NB
PDZ3:INT	NB	NB	NB	NB	NB
PDZ4:INT	NB	NB	NB	NB	NB
PDZ3 K1040A:CTN	0.9 ± 0.01	12.5 ± 0.5	−6.7 ± 0.02	−6.7 ± 0.4	−0.05 ± 0.5
PDZ3 H1071A:CTN	NB	NB	NB	NB	NB

CTN, C-terminal sequence LRLESSII; INT, Internal sequence EMYYSTAVTGNI. All affinities were measured using isothermal titration calorimetry, with *K*_D_ values given in µM as a mean of three independent experiments with SD. NB = ‘no binding’, NT = ‘not tested’ and N denotes stoichiometry.

### The crystal structures of SCRIB PDZ3:NS5_CTN and SCRIB PDZ3

**Figure 3.**
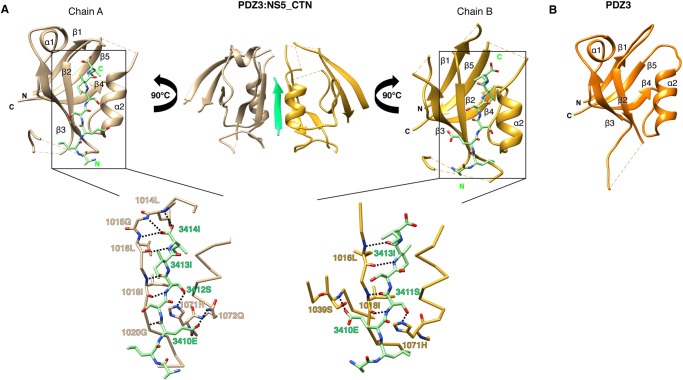
The crystal structures of the SCRIB PDZ3 and SCRIB PDZ3:NS5_CTN complex. (**A**) SCRIB PDZ3 chain A (tan) and chain B (gold) are shown as a cartoon with NS5_CTN peptides (green) represented as sticks. Bottom panel: Close up view of interactions in the SCRIB PDZ3:NS5_CTN interfaces. Interactions are denoted as black dotted lines. (**B**) SCRIB PDZ3 (orange) shown as a cartoon. Interactions are denoted as black dotted lines.

To understand the interactions of SCRIB PDZ domain interactions with NS5 PBM sequences in more detail we determined crystal structures of SCRIB PDZ3:NS5_CTN complexes as well as a structure of SCRIB PDZ3 apo (Figure 3, Table 2).

**Table 2:** X-ray data collection and refinement statistics

	PDZ3:NS5_CTN	PDZ3
**Data collection**		
Space group	R 3 2	P 2_1_ 2_1_ 2_1_
No. of molecules in AU	two molecules of PDZ3 + one molecule of NS5_CTN	two molecules of PDZ3
Cell dimensions		
*a*, *b*, *c* (Å)	69.96 69.96 201.60	36.88 60.89 64.90
*α*, *β*, *γ* (°)	90.00 90.00 120.00	90 90 90
Wavelength (Å)	0.95	0.95
Resolution (Å)	29.96–2.05 (2.12–2.05)	32.45–1.84 (1.91–1.844)
*R*_sym_ or *R*_merge_	0.046 (0.63)	0.031 (0.36)
I/σI	6.91 (0.80)	9.16 (1.25)
CC (1/2)	0.99 (0.51)	0.99 (0.88)
Completeness (%)	99.25 (99.84)	99.63 (99.21)
Redundancy	1.97 (1.97)	2.0 (2.0)
Wilson B-factor	39.77	25.42
**Refinement**		
No. of reflections	24 260 (2401)	13 080 (1259)
*R*_work_/*R*_free_	0.24/0.29	0.24/0.29
No. of non-hydrogen atoms		
Protein	1302	1172
Water	62	40
B-Factors		
Protein	46.14	34.12
Water	47.62	35.89
r.m.s.d.		
Bond lengths (Å)	0.013	0.009
Bond angle (°)	1.34	0.95
Ramachandran plot (%)		
Favored	97.35	100.00
Allowed	2.65	0
Disallowed	0	0

Values in parentheses are for the highest resolution shell.

The complex of SCRIB PDZ3:NS5_CTN revealed an unexpected stoichiometry, where a single NS5_CTN peptide was bound in an antiparallel manner in the canonical PDZ binding groove of a single SCRIB PDZ3 molecule (Chain A), with a second SCRIB PDZ3 molecule (Chain B) engaging the same peptide via its β2 strand (Figure 3A). In this 2:1 complex an extended β-sheet between Chain A β2-NS5_CTN-Chain B β2 is formed. Examination of the SCRIB PDZ3 molecule engaging NS5_CTN using the canonical ligand binding groove reveals that eight hydrogen bonds are formed upon binding, including a double bond between Ser3412^NS5^–Ile1018^PDZ3^, and single bonds with Ile3414^NS5^–Leu1014^PDZ3^, Ile3414^NS5^–Gly1015^PDZ3^, Ile3414^NS5^–Leu1016^PDZ3^, Glu3410^NS5^–Gln1072^PDZ3^ and Ile3409^NS5^–His1023^PDZ3^ as well as Ser3412^NS5^–His1071^PDZ3^ (Figure 3C). The non-canonical interaction between a second SCRIB PDZ3 domain and NS5_CTN involves two hydrogen bonds with Ile3413^NS5^–Leu1016^PDZ3^ and Ser3412^NS5^–Ile1018^PDZ3^, as well as a single bond between Glu3410^NS5^–Ser1039^PDZ3^ at β3 and Ser3411^NS5^–His1071^PDZ3^ (Figure 3C).

**Figure 4.**
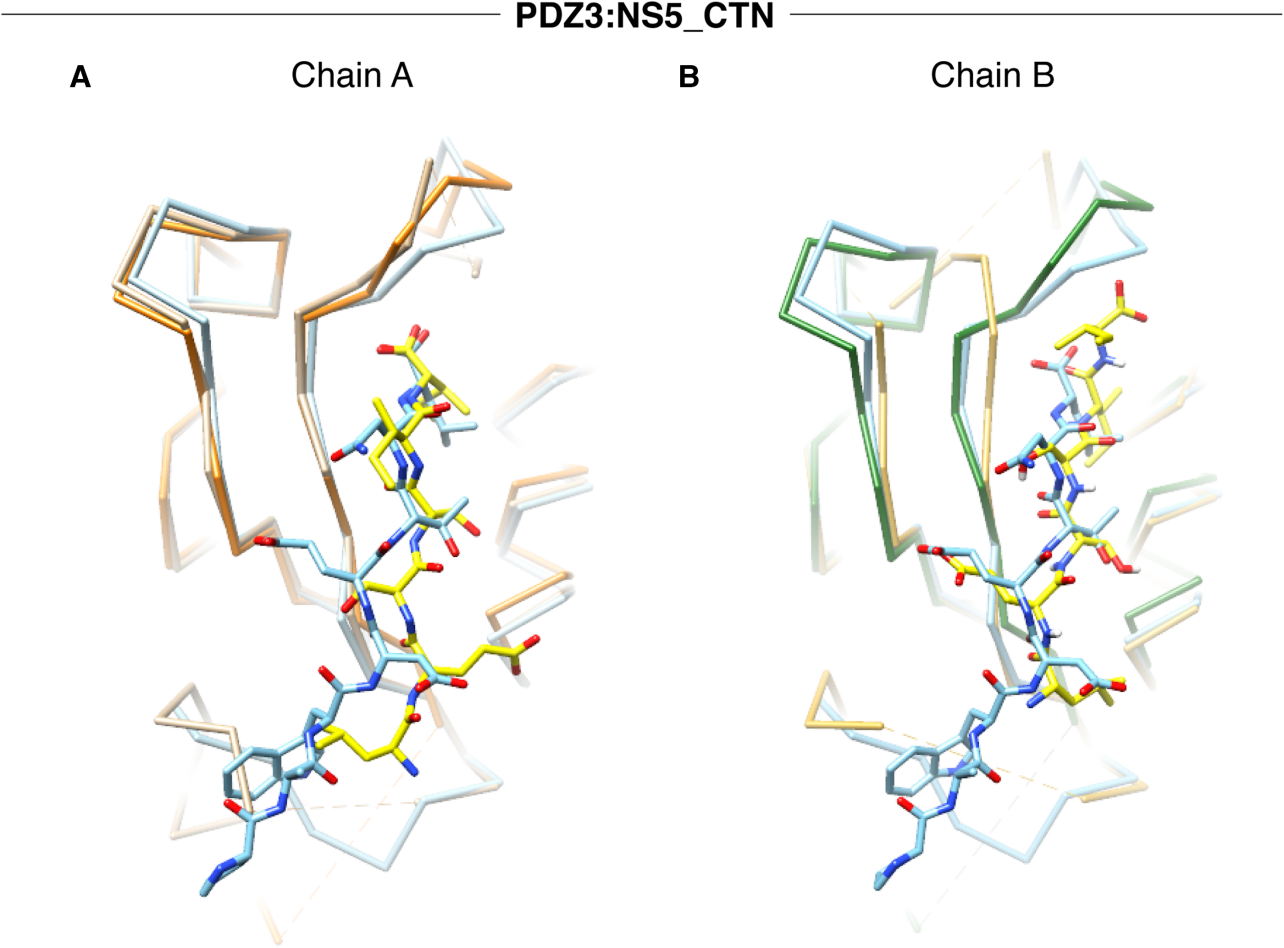
Close up view of superimposition of SCRIB PDZ3:NS5 complex against previously determined Scrib PDZ structures bound to host PBM peptides, represented as chain trace and sticks. (**A**) SCRIB PDZ3:NS5_CTN Chain A (tan:yellow) superimposed with SCRIB PDZ3:β-PIX (light blue, PDB ID 5VW1) and SCRIB PDZ3apo (forest green). (**B**) SCRIB PDZ3:NS5_CTN Chain B (goldenrod:yellow) superimposed with SCRIB PDZ3:β-PIX (light blue, PDB ID 5VW1) and SCRIB PDZ3apo (forest green).

When the SCRIB PDZ3:β-PIX (PDB ID: 5VWI) complex is superimposed on our PDZ3:NS5_CTN complex, an RMSD of 0.78 Å for Chain A and 0.75 Å for Chain B (Figure 4) over the Cα backbone atoms is revealed. β-PIX engages with SCRIB PDZ3 via hydrogen bonds formed by Ser1017^PDZ3^–Asn645^β-PIX^, Ser1026^PDZ3^–Trp641^β-PIX^, Ser1039^PDZ3^–Glu643^β-PIX^ and H1071^PDZ3^–T644^β-PIX^. In the Scrib PDZ3:NS5_CTN complex, His1071^PDZ3^ contacts Ser3412^NS5^ in the −2 position in the canonical manner observed in many other PDZ domain complex structures [21–24]. Interesting, Glu3410^NS5^ is able to contact Gln1072^PDZ3^ whereas in the PDZ3: β-PIX complex the equivalent Asp642^β-PIX^ is too short to reach Gln1072^PDZ3^. The Ser1039^PDZ3^–Glu643^β-PIX^ interaction is recapitulated in a more unconventional configuration, with the Ser1039^PDZ3^ being provided by chain B which binds NS5 in a non-canonical manner.

Binding of NS5_CTN in a non-canonical manner requires no significant change in the SCRIB PDZ3 domain fold, with a superimposition of both SCRIB PDZ3 chains yielding an RMSD of 1.15 Å in Chain A and 1.35 Å in Chain B (Figure 4) over the Cα backbone atoms. Scrib PDZ3 apo adopts the typically PDZ domain fold, and revealed no distinct differences in overall fold to other monomeric SCRIB PDZ1 (PDB ID: 5VWC) and SCRIB PDZ2 (PDB ID: 7JO7) structures, as shown by RMSD values of 1.03 Å and 1.15 Å over the Cα backbone atoms for superimpositions with Scrib PDZ1 and 2, respectively (Figure 4B). Furthermore, all side chain rotamers of residues involved in the canonical ligand binding groove are identical with those found in PDZ3:NS5_CTN, including His1071^PDZ3^. No major changes in B-factor distribution for backbone atoms are observed upon NS5_CTN binding.

**Figure 5.**
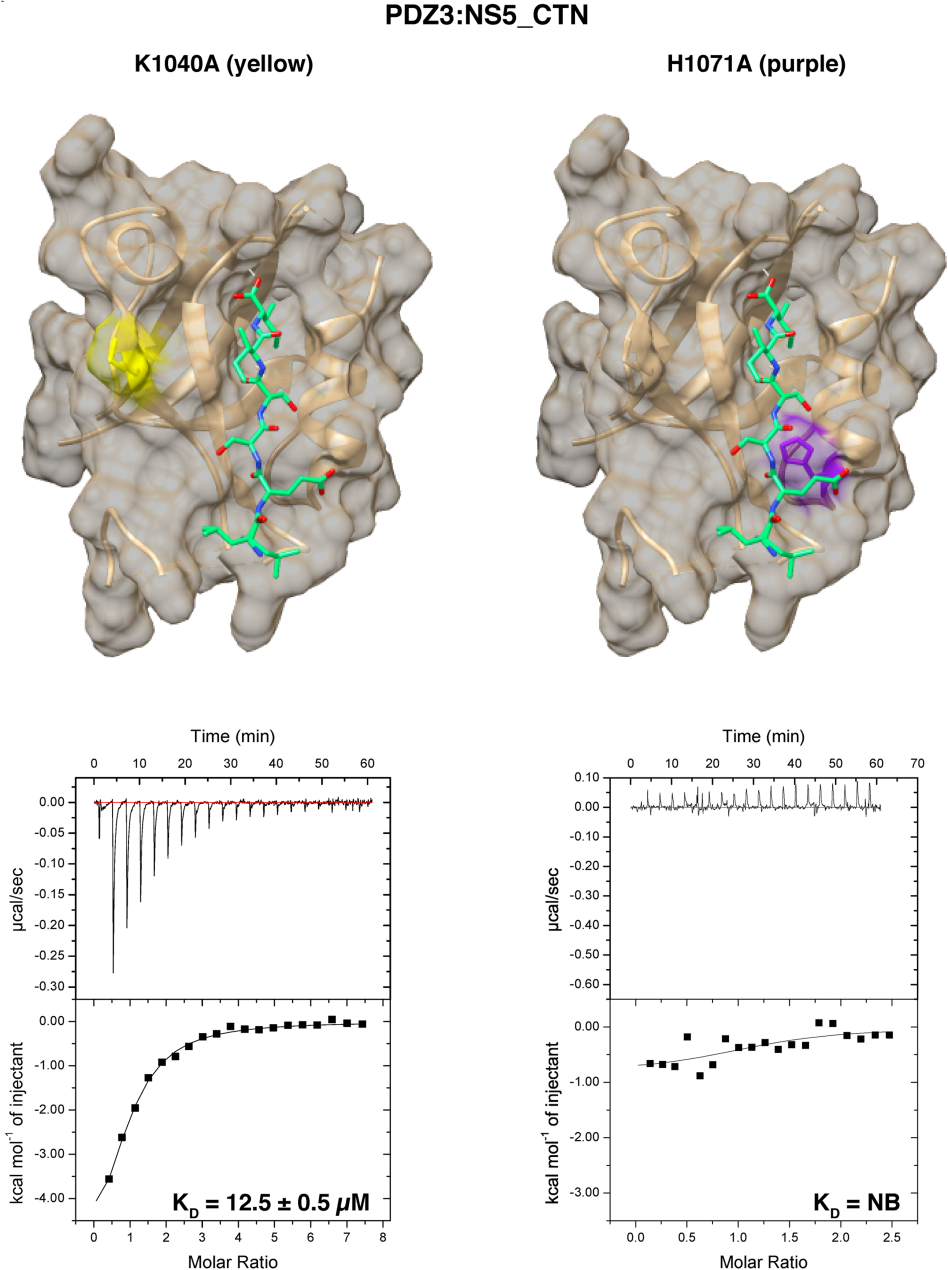
Location of Scribble PDZ domain mutations. Mutated residues are coloured on a surface representation of the relevant Scribble PDZ domain constructs for SCRIB PDZ3 K1040A (yellow) and SCRIB PDZ3 H1071A (purple). Binding isotherms of isolated mutant Scribble PDZ domain interactions with NS5_CTN peptide. Each profile is represented by a raw thermogram, and a binding isotherm fitted with a one-site binding model (bottom panels). *K*_D_: dissociation constant; ±: standard deviation; NB: no binding. Each value was calculated from at least three independent experiments.

We next performed site-directed mutagenesis to validate the binding of NS5_PBM peptides to SCRIB PDZ domain. Our crystal structure of SCRIB PDZ3 bound to PBM sequences revealed an interaction with a conserved His residue. Mutation of SCRIB PDZ3 H1071 to Ala ablates binding, whereas mutation of SCRIB PDZ3 K1040, which is not involved in NS5 engagement, did not significantly impact binding affinity (Table 1 Figure 5). This supports the notion that SCRIB PDZ3 H1071 plays an important role in the NS5 CTN engagement. Interestingly, mutation of K1040 resulted in a notable change in ΔH and −TΔS between SCRIB PDZ3:NS5_CTN (−1.1 ± 0.4 and 5.3 ± 0.4 kcal mol^−1^) and SCRIB PDZ3 K1040A:NS5_CTN (−6.7 ± 0.4 and −0.05 ± 0.5 kcal mol^−1^), suggesting that the SCRIB PDZ3 K1040 position significantly impacts the enthalpic contribution to NS5_CTN binding. However, the *K*_D_ difference of SCRIB PDZ3:CTN (18.4 ± 1.3 µM) and SCRIB PDZ3 K1040A:CTN (12.5 ± 0.5) is modest. To ensure that the mutants were correctly folded, CD spectroscopy was performed on mutant proteins, which revealed spectra simular to the corresponding wild-type PDZ domain spectra with comparable proportions of secondary structure elements (Supplementary Figure S2). Both wild-type and mutant spectra displayed the characteristic features of a double minimum at ∼208 nm and 225 nm and maximum at ∼200 nm indicative of a fold containing mixed alpha/beta secondary structure elements, suggesting that they were similarly folded [25].

## Discussion

Scribble is an important regulator of cell polarity and acts as a scaffolding protein to mediate a diverse set of cellular signalling pathways via its four PDZ domains [2]. Scribble interacts with a diverse set of interactors including host proteins β-PIX, Vangl2, APC and Gukh [23, 24, 26, 21 22], as well as virus encoded proteins such as influenza A H1N1 NS1, HTLV-1 Tax, HPV E6 and TBEV NS5 [10, 22, 27–30, 21 31]. As a result, multiple cellular ligands may compete for Scribble binding with each other as well as with a virus encoded ligand, and thus only a certain subset of potential interactors are able to bind at any given time. However, the molecular details of such selectivity and specificity remain to be fully described. This is in part due to additional factors that affect Scrib PDZ domain ligand recognition beyond the amino acid sequence of the PBM motif, including phosphorylation state [23, 32] and possible occlusion of ligand binding groove for a given PDZ domain in the context of a larger multi-domain protein [33–35], as well as competitive binding due to the presence of multiple interactors at the same time [7]. To understand how virus encoded proteins engage Scribble's PDZ domains and perturb Scribble mediated cellular signalling pathways, we examined the interactions of TBEV NS5 with Scribble using affinity measurements and high resolution crystal structures to gain insight into TBEV driven host cell manipulation. These affinity measurements will serve as the first step to examine how viral Scribble interactors compete with endogenous host cell interacting proteins.

Viruses have adapted to target polarity signalling in general [36] and Scribble in particular to engineer a more supportive cellular environment for viral replication [5]. This is underscored by the observation that the presence of a PBM in a viral protein has been linked to increased virulence during viral pathogenesis [27, 29, 37]. Within the *flaviviridae*, the neuroinvasive TBEV has been shown to have a high fatality rate of 30%, and it has been postulated that TBEV targets Scribble to evade the innate immune responses [10, 12, 19, 38].

Previous studies suggested that the interactions between Scribble and NS5 were solely via an internal PBM as shown in a yeast-2-hybrid assay, which revealed binding to SCRIB PDZ4, whereas the canonical C-terminal PBM appeared to not be involved in Scribble interactions [9]. We now show the canonical C-terminal PBM of NS5 does bind SCRIB PDZ3 with 18.4 ± 1.3 µM *K*_D_ affinity (Table 1, Figure 2). Unexpectedly, in our hands the previously identified internal NS5 PBM EMYYS (INT, Figure 1A) did not bind to any of Scribble's PDZ domains when subjected to ITC analysis (Table 1, Figure 2). Examination of the full length NS5 protein structure revealed that this sequence is located in the middle of a β-sheet, likely preventing the exposure of the PBM residues for canonical binding to PDZ domains, suggesting that this novel PBM sequence is unlikely to be functionally relevant [39] (Figures 1 and 2). We note that we examined the EMYYS PBM in the context of a 12-mer peptide (EMYYSTAVTGNI), whereas the initial report featured the sequence GGGLVRTPGSRNSTHEMYYS. The presence of additional N-terminal residues may underly the differential interaction pattern we observed in particular with Scrib PDZ4.

Unexpectedly, when determining the crystal structure of the SCRIB PDZ3:NS5_CTN complex we observed a heterotrimer with a 2 : 1 stoichiometry comprising only a single NS5_CTN chain (Figure 3A). Here, the second SCRIB PDZ3 protomer bound the single NS5_CTN chain in a manner that creates an extended β-sheet (Figure 3A). Previously SCRIB PDZ3 had only been shown to form 1 : 1 heterodimers with PBM peptides via the canonical PDZ binding groove. However, other PDZ domains have been shown to feature bound interactors as a component of an extended β-sheet, as observed for nNOS PDZ or Shank PDZ [18]. Examination of the SCRIB PDZ3:NS5_CTN interaction using ITC demonstrated a 1 : 1 stoichiometry (Table 1), suggesting that the binding of the second protomer in a non-canonical manner is likely due to crystallographic packing and not biologically relevant.

It is uncommon for an interacting PBM sequence to only bind to a single PDZ domain of SCRIB as seen with NS5_CTN only binding to PDZ3 (Table 1, Figure 2), with most SCRIB interacting PBMs binding to multiple PDZ domains including β-PIX, Vangl2, APC, MCC and Gukh [2, 23, 24, 26, 21 22]. However PBMs belonging to GUGY1A2, TRIP6 and LPP appear to engage only a single SCRIB PDZ domain [40, 41]. Although Werme et al. had reported that SCRIB PDZ4 was the sole interactor for NS5, we were unable to confirm this with our ITC measurements [10] (Table 1, Figure 2). This could be due to the different experimental methodologies employed in our study; Werme and colleagues relied on a yeast-2-hybrid assay and used sequences of different length as well as N- and C-terminal extensions, whereas our investigation focussed on using recombinantly expressed and purified individual PDZ domains in conjunction with quantitative affinity measurements to probe PDZ-PBM interactions [10]. However, we note that in the case of the SCRIB PDZ3:NS5 interaction via the C-terminal PBM, we were able to reveal the details of this interaction crystallographically. The lack of a SCRIB PDZ4 interaction raises the question of the role of PDZ4 for Scribble signalling and interactions. Other ligands such as ZO-2, DLC3, GluN2A, GluN2B and NOS1AP have been reported to bind to SCRIB PDZ4 as well as to other Scrib PDZ domains [42–45]. SCRIB PDZ4 may play a more regulatory role by modulating interdomain interactions rather than a direct ligand-domain role, as seen in the SCRIB PDZ3/4 tandem domain structure (PDZ34) [46]. In that case, the interacting peptide primarily used the αB/βB pocket of PDZ3, with PDZ4 effectively used to expand the binding grove to create an elongated site across the SCRIB PDZ34 supramodule [46], thus providing another level of structural regulation within the PDZ domains.

In summary, we show that TBEV NS5 C-terminal PBM binds SCRIB PDZ3 in order to interact with Scribble. Our findings provide a structural basis for Scribble PDZ domains and TBEV NS5 interactions and will enable more detailed structure-guided investigations including increased ligand specificity via protein engineering to dissect the pathogenesis of TBEV and the role of cell polarity signalling in more detail.

## Materials and methods

### Protein expression and purification

Human Scribble (SCRIB) (Uniprot accession number: Q14160) PDZ1 (728–815); PDZ2 (860–950); PDZ3 (1002–1094); PDZ4 (1099–1203)) domain proteins as well as mutant SCRIB PDZ domain proteins were subcloned into the bacterial expression vector pGEX-6P1, and expressed and purified as previously described [21]. Briefly, protein overexpression was performed using *Escherichia coli* BL21 (DE3) pLysS cells (BIOLINE) in super broth supplemented with 200 µg/ml ampicillin (AMRESCO) using auto-induction media (10 mM Tris–Cl pH7.6, 100 mM NaCl, 1 mM MgSO_4_, 0.2% (w/v) D-lactose, 0.05% (w/v) glucose, 0.5% (v/v) glycerol) [47] at 37°C until the optical density at 600 nm (OD_600_) reached 1.0 before transferring cultures to 16°C for 24 h for protein expression.

Bacterial cells were harvested by centrifugation and lysed in the presence of deoxyribonuclease I (Sigma–Aldrich) from bovine pancreas using TS Series 0.75 kw model cabinet (Constant Systems Ltd.) at 25 kPsi or a Fastprep®-24 (MP Biomedicals) using lysing matrix B for 20 s. Lysates were clarified by centrifugation at 20 000×***g*** for 20 min using an Avanti® J-E (Beckman Coulter). The resulting supernatant was filtered using Millex-GP syringe filter unit 0.22 µM (Merck Millipore) prior to loading onto equilibrated columns for affinity purification. Glutathione-S-transferase (GST) tagged recombinant proteins were captured using glutathione sepharose 4B (GE Healthcare) in buffer A (50 mM Tris–Cl pH 8.0, 150 mM NaCl and 1 mM EDTA) and eluted with buffer A supplemented with 20 mM reduced l-glutathione. Recombinant proteins were subsequently dialysed into buffer A to remove glutathione for the pull down assay. GST tagged PDZ domains were cleaved on-column with HRV 3C protease to remove the GST tag. All recombinant PDZ domain proteins were then subjected to size-exclusion chromatography using a Superdex S75 10/300 column mounted on an AKTA Pure and equilibrated in 20 mM HEPES pH 7.2 and 150 NaCl, where they eluted as single peaks. PDZ2, PDZ3 and PDZ4 lacked Tryptophan residues, requiring concentration quantification via their peptide bond absorption at 205 nm using the Scopes method [48]. The absorbance was calculated as their respective concentration at a wavelength of 205 nm using the NanoDrop spectrophotometer. The concentration of SCRIB PDZ1, which contained a Tryptophan residue, was determined at 280 nm absorbance via NanoDrop 2000/2000c Spectrophotometer (Thermo Fisher Scientific). UV absorption values were then substituted into the following equation; Concentration (mg/ml) = A280/E, where E represent the extinction coefficient.

### Peptides

The following peptides were commercially synthesised using solid phase synthesis with Fmoc-protection strategy with subsequent trifluoroacetic acid removal (Genscript) and used in this study: TBEV TBEV NS5_INT: EMYYSTAVTGNI (amino acids 2730–2741), TBEV NS5_CTN: LRLESSII (amino acids 3407–3414) (UniProt accession code Q01299) (Figure 1A). Superpeptide (RSWFETWV) was originally identified via the screening of a phage-displayed peptide library, and was shown to harbor affinity towards a wide range of different PDZ domains and is used as a positive control [42] (Supplementary Figure S3). All peptides were unmodified.

### Circular dichroism (CD) spectroscopy

All proteins were diluted to 0.150 mg/ml in phosphate buffer (50 mM phosphate pH 7.0) and subjected to CD spectroscopy using the AVIV model 420 CD spectrometer at a wavelength from 190–260 nm with sample entry every 1 nm and an averaging time of 4 s at 25°C. Data were processed using the AVIV Biomedical software and plotted using Excel. Deconvolution of spectra to determine secondary structure composition was performed using Bestsel [49].

### Isothermal titration calorimetry

SCRIB PDZ1 to PDZ4 proteins were prepared in 20 mM HEPES pH 7.2 and 150 NaCl at a final concentration of 75 µM, with peptides prepared in the same buffer at a concentration of 900 µM. Peptide concentrations were calculated based on the dry peptide weight after synthesis, and were commercially synthesised (Genscript).

Independent ITC experiments were conducted using a MicroCal iTC200 System (GE Healthcare) at 25°C at a stirring speed of 750 rounds-per-minute. A total of 20 injections of 2 µl each and a spacing of 180 s were titrated into the 200 µl protein sample (except for injection 1 which was 0.4 µl), as previously described [21]. Each interaction was measured 3 times, with the results averaged to calculate binding affinities, stoichiometries and graphical interpretations with their respective standard deviation (Table 1, Figure 2, Supplementary Figure S5). The differential power change caused by heat reactions from each interaction was processed with the Origin 7.0 software (OriginLab Corporation) using ‘one-site binding model’.

### Crystallisation and structure determination

SCRIB PDZ domain:NS5 peptide complexes were prepared using a protein:peptide molar ratio of 1 : 4 as previously prescribed [22]. Protein used are in 20 mM HEPES pH 7.2 and 150 NaCl. Concentrated protein complex samples were subjected to high-throughput crystallisation screening using a Gryphon LCP (Art Robbins Instruments) with 0.2 µl of protein sample and 0.2 µl reservoir in the drop. The following sparse matrix screens were used; JCSG-plus: HT-96 sparse matrix screen (Molecular Dimensions), PACT Premier HT-96 sparse matrix screen (Molecular Dimensions), Structure Screen 1 + 2 sparse matrix screen (Molecular Dimensions) and Salt Rx sparse matrix screen (Hampton Research). Optimisation of identified crystallization conditions was performed in 24-well sitting drop plates (Hampton Research) at 20°C with a drop size of 2 µl comprising 1 µl of protein sample and 1 µl of reservoir solution. Crystals were mounted on nylon and copper loops (MiTGen) and diffraction data collected at the Australian Synchrotron using the MX2 beamline equipped with the Eiger 16M detector with an oscillation range 0.1° per frame with a wavelength of 0.9537, integrated using XDS [50] and scaled using AIMLESS [51].

Crystals of PDZ3:NS5_CTN were grown in 0.2 M Sodium nitrate, 0.1 M Bis-Tris propane pH 7.5, 20% w/v PEG 3350 and were flash cooled at −173°C in mother liquor. PDZ3:NS5_CTN complex formed single dome-shaped crystals beloning to the space group R32 with *a* = 69.96 Å, *b* = 69.96 Å, *c* = 201.67 Å, *α* = 90.00°, *β* = 90.00°, *γ* = 120° in the trigonal crystal system at a resolution of 2.05 Å (Table 2). Molecular replacement was carried out using PHASER [52] with the previously solved structure of Scrib PDZ3:β-PIX (PDB ID: 5VWI) as a search model. PDZ3:NS5_CTN crystals contained two molecules of PDZ3 and 1 of NS5_CTN peptide in the asymmetric unit, with 47.5% solvent content and final TFZ and LLG values of 15.10 and 315.19, respectively. The final model of PDZ3:NS5_CTN was built manually over several cycles using Coot [53] and refined using PHOENIX [54] with final *R*_work_/*R*_free_ of 0.24/0.29, with 97.35% of residues in the favoured region of the Ramachandran plot and 0% of rotamer outliers.

Crystals of SCRIB PDZ3 were grown in 1.5 M Sodium nitrate, 0.1 M Sodium acetate trihydrate pH 4.6 and were flash cooled at −173°C in mother liquor. Crystals of SCRIB PDZ3 grew as cluster sof plates belonging to the space group P2_1_2_1_2_1_ with *a* = 36.88 Å, *b* = 60.89 Å, *c* = 64.90 Å, *α* = 90.00°, *β *= 90.00°, *γ *= 90.00° in the monoclinic crystal system at a resolution of 1.84 Å (Table 2). Molecular replacement was carried out using PHASER [52] with the previously solved structure of SCRIB PDZ1:β-PIX (PDB ID: 5VWK) as a search model. SCRIB PDZ3 crystals contained two molecules of SCRIB PDZ3 in the asymmetric unit, with 52.70% solvent content and final TFZ and LLG values of 10.4 and 160.55, respectively. The final model of SCRIB PDZ3 was built manually over several cycles using Coot [53] and refined using PHENIX [54] with final *R*_work_/*R*_free_ of 0.24/0.29, with 100% of residues in the favoured region of the Ramachandran plot and no rotamer outliers.

## Data Availability

Coordinate files have been deposited in the Protein Data Bank under the accession codes 7QSA and 7QSB. All raw diffraction images were deposited on the SBGrid Data Bank [55] using their PDB accession codes.

## Abbreviations

DENV: Dengue virus
GST: Glutathione-S-transferase
ITC: isothermal titration calorimetry
MTase: methyltransferase
PBMs: PDZ binding motifs
TBEV: Tick-born encephalitis virus
WNV: West Nile Virus
