# Supplementary material for: Molecular basis of Tick Born encephalitis virus NS5 mediated subversion of apico-basal cell polarity signalling
Source: Biochem J. 2022 Jun 22;479(12):1303–15. doi: 10.1042/BCJ20220037 (PMC9317960; doi:10.1042/BCJ20220037)
Supplement: Supplementary Material [file BCJ-479-1303-s1.pdf]

Supplementary material

Supplementary Figure Legends

Figure S1. Thermodynamic analysis of SCRIB PDZ domain binding to NS5 PBM peptides. The contribution of -ΔH (kcal·mol−1) and TΔS (kcal mol−1 K−1) to the binding of NS5_CTN are shown. Each of the values were calculated from at least three independent experiments. NS5_CTN sequence is LRLESSII.

Figure S2. Circular dichroism spectroscopy of wild type and mutant Scribble PDZ1 and PDZ3 mutants. (A)-(E) Circular dichroism spectra recorded for wild type and mutant Scribble PDZ1,2,3 and 4 domains indicated that there were no major spectral differences between the proteins, suggesting that they were similarly folded, with mutations not leading to unfolding of the PDZ domains.

Figure S3. Interactions of Scribble PDZ domains with superpeptide. Affinities were measured using isothermal titration calorimetry (ITC) and the raw thermograms are shown. KD values (in µM) are the means of 3 experiments ± SD.

Figure S4. Raw ITC thermograms of the interactions of Scribble PDZ constructs with NS5 PBM peptides. Affinities were measured using isothermal titration calorimetry (ITC). **(A)** Interactions of wild-type Scribble PDZ1, 2, 3 and 4 domains with NS5_INT or NS5_CTN peptides. **(B)** Interactions of mutant Scribble PDZ3 domains with NS5_CTN peptide.

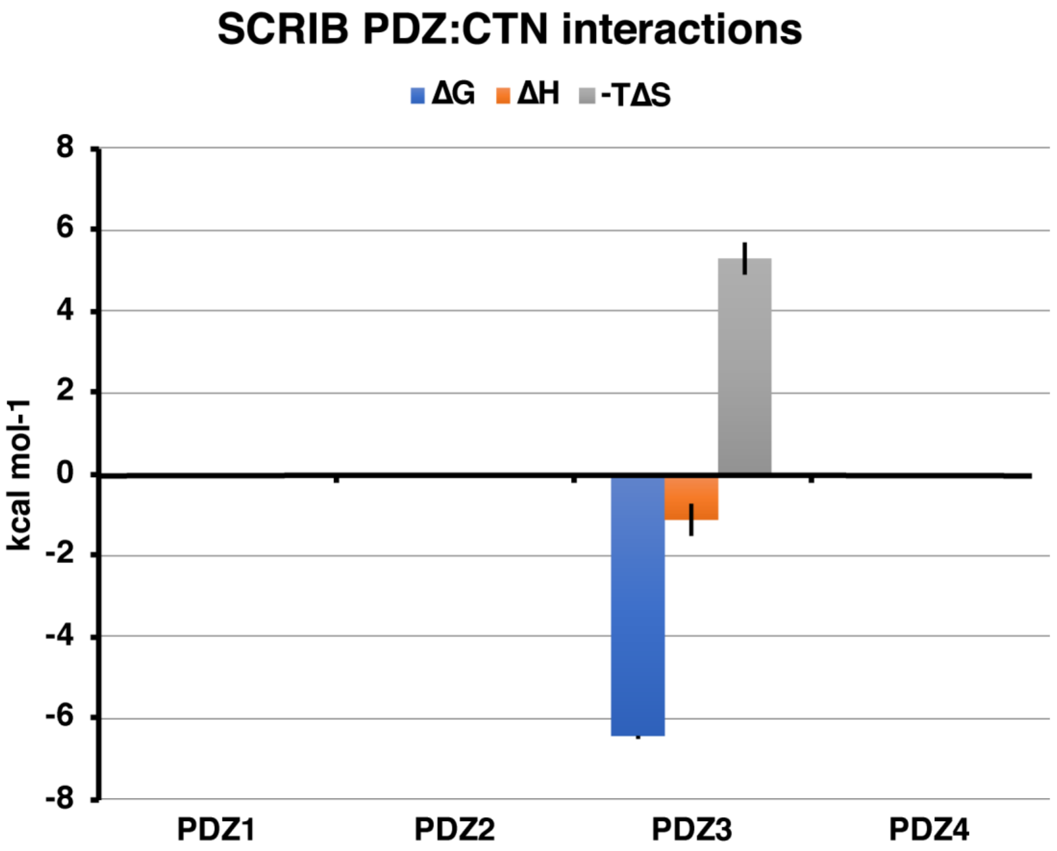

SCRIB PDZ:CTN interactions
ΔG
ΔH
-TΔS
kcal mol-1
8
6
4
2
0
-2
-4
-6
-8
PDZ1
PDZ2
PDZ3
PDZ4

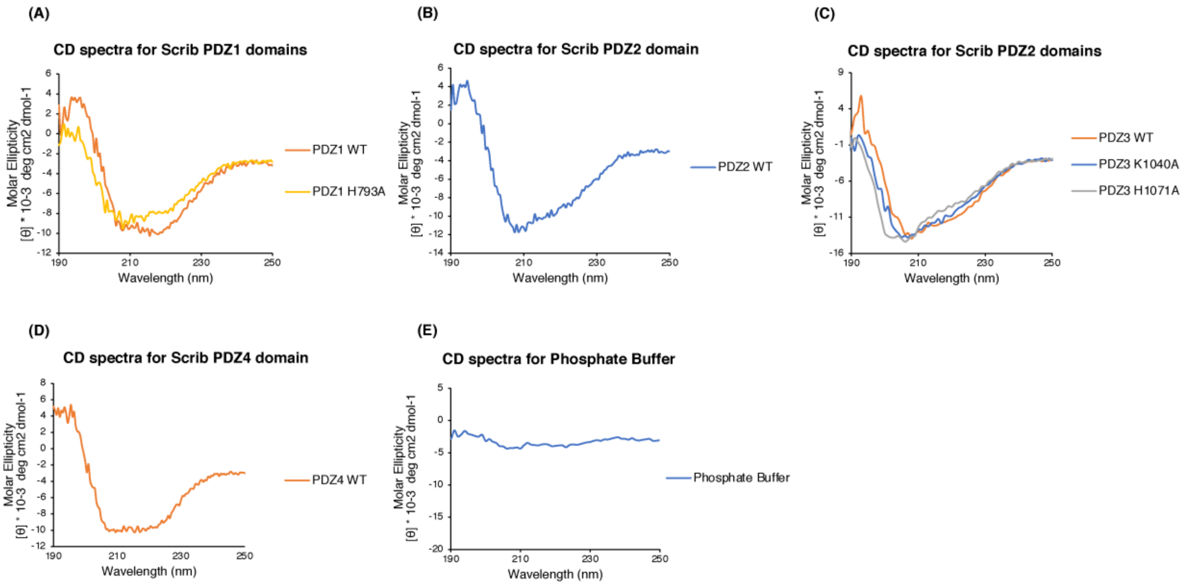
(A)
CD spectra for Scrib PDZ1 domains
Molar Ellipticity
[θ] * 10-3 deg cm2 dmol-1
Wavelength (nm)
PDZ1 WT
PDZ1 H793A
(B)
CD spectra for Scrib PDZ2 domain
Molar Ellipticity
[θ] * 10-3 deg cm2 dmol-1
Wavelength (nm)
PDZ2 WT
(C)
CD spectra for Scrib PDZ2 domains
Molar Ellipticity
[θ] * 10-3 deg cm2 dmol-1
Wavelength (nm)
PDZ3 WT
PDZ3 K1040A
PDZ3 H1071A
(D)
CD spectra for Scrib PDZ4 domain
Molar Ellipticity
[θ] * 10-3 deg cm2 dmol-1
Wavelength (nm)
PDZ4 WT
(E)
CD spectra for Phosphate Buffer
Molar Ellipticity
[θ] * 10-3 deg cm2 dmol-1
Wavelength (nm)
Phosphate Buffer

(A)

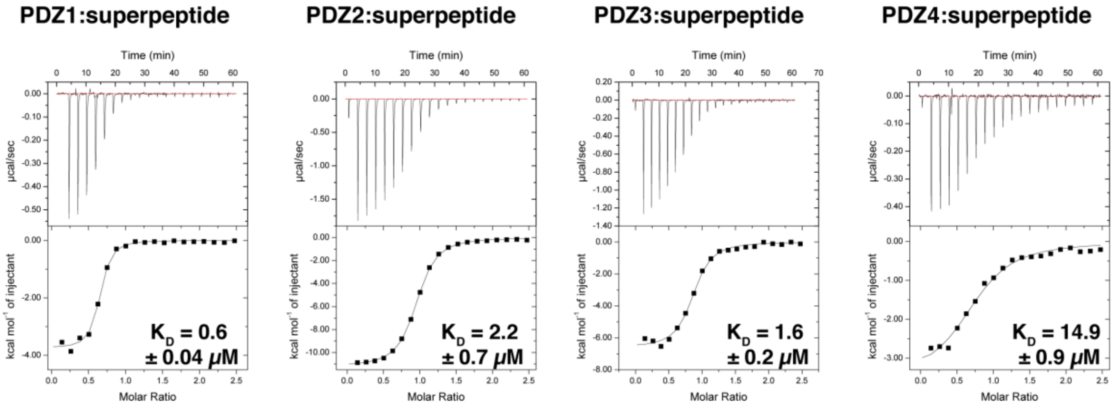
PDZ1:superpeptide
PDZ2:superpeptide
PDZ3:superpeptide
PDZ4:superpeptide
Time (min)
µcal/sec
kcal mol-1 of injectant
Molar Ratio
K_D = 0.6 ± 0.04 µM
K_D = 2.2 ± 0.7 µM
K_D = 1.6 ± 0.2 µM
K_D = 14.9 ± 0.9 µM

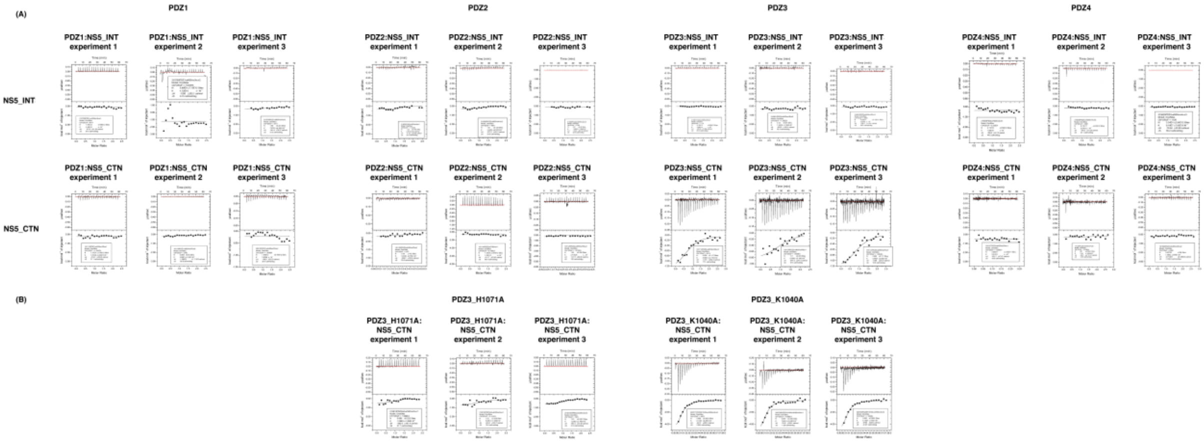
(A)
PDZ1
PDZ2
PDZ3
PDZ4
NS5_INT
NS5_CTN
PDZ1:NS5_INT experiment 1
PDZ1:NS5_INT experiment 2
PDZ1:NS5_INT experiment 3
PDZ2:NS5_INT experiment 1
PDZ2:NS5_INT experiment 2
PDZ2:NS5_INT experiment 3
PDZ3:NS5_INT experiment 1
PDZ3:NS5_INT experiment 2
PDZ3:NS5_INT experiment 3
PDZ4:NS5_INT experiment 1
PDZ4:NS5_INT experiment 2
PDZ4:NS5_INT experiment 3
PDZ1:NS5_CTN experiment 1
PDZ1:NS5_CTN experiment 2
PDZ1:NS5_CTN experiment 3
PDZ2:NS5_CTN experiment 1
PDZ2:NS5_CTN experiment 2
PDZ2:NS5_CTN experiment 3
PDZ3:NS5_CTN experiment 1
PDZ3:NS5_CTN experiment 2
PDZ3:NS5_CTN experiment 3
PDZ4:NS5_CTN experiment 1
PDZ4:NS5_CTN experiment 2
PDZ4:NS5_CTN experiment 3
(B)
PDZ3_H1071A
PDZ3_K1040A
PDZ3_H1071A: NS5_CTN experiment 1
PDZ3_H1071A: NS5_CTN experiment 2
PDZ3_H1071A: NS5_CTN experiment 3
PDZ3_K1040A: NS5_CTN experiment 1
PDZ3_K1040A: NS5_CTN experiment 2
PDZ3_K1040A: NS5_CTN experiment 3

CERTIFICATE OF ANALYSIS

Product Name	TBEV_NS5_Int_PBM3
Order ID	U877LEH160_7
Lot No.	U877LEH160-7/PE6405
Sequence	LRLESSII
Modification	N/A
Length	8AA
Storage	-20°C
Recommended Solvent*	dimethyl sulfoxide(Analytical grade)
comments	TFA salt

Test Items	Specifications	Results
Molecular Weight	Theoretical MW: 930.11	Consistent
HPLC purity	≥95.0%	97.1%
Appearance	White lyophilized powder	Conforms
Gross Weight	14 mg	2*7mg

*Note: Above recommended solvents for reference only. If there is any request for detailed dissolution conditions,we suggest you choose our 'Peptide Solubility Test Service'.

Caution:
For laboratory or further manufacturing use only. Not intended for household use. If you have any questions about the Certificate of Analysis, please contact our customer service representative at 1-877-436-7274 (Toll-Free), or 1-732-885-9188.

Certified by: Gary Chen Date:08-29-2019
Peptide Production Director

Thank you for your patronage to our Peptide services! To maintain this working relationship, we shall be grateful if you can add our webpage URL into your lab website. As a token of appreciation, you will be rewarded by 1,000 EZcoupon™ points. For more information, please contact us by e-mail at web@genscript.com

Sample Name: TBEV_NS5_Int_PBM3
Sample ID: U877LEH160-7
Time Processed :0:47:10
Month-Day-Year Processed :08/25/2019

Pump A : 0.065% trifluoroacetic in 100% water (v/v)
Pump B : 0.05% trifluoroacetic in 100% acetonitrile (v/v)
Total Flow:1 ml/min
Wavelength:220 nm

Time	Unit	Command	Value	Comment
0.01	Pumps	Pump A B.Conc	5	
25.00	Pumps	Pump A B.Conc	65	
25.01	Pumps	Pump A B.Conc	95	
27.00	Pumps	Pump A B.Conc	95	
27.01	Pumps	Pump A B.Conc	5	
35.00	Pumps	Pump A B.Conc	5	
35.01	Controller	Stop		

<<Column Performance>>
<Detector A>
Column : Inertsil ODS-3 4.6 x 250 mm
Equipment: GK1101009

Chromatogram

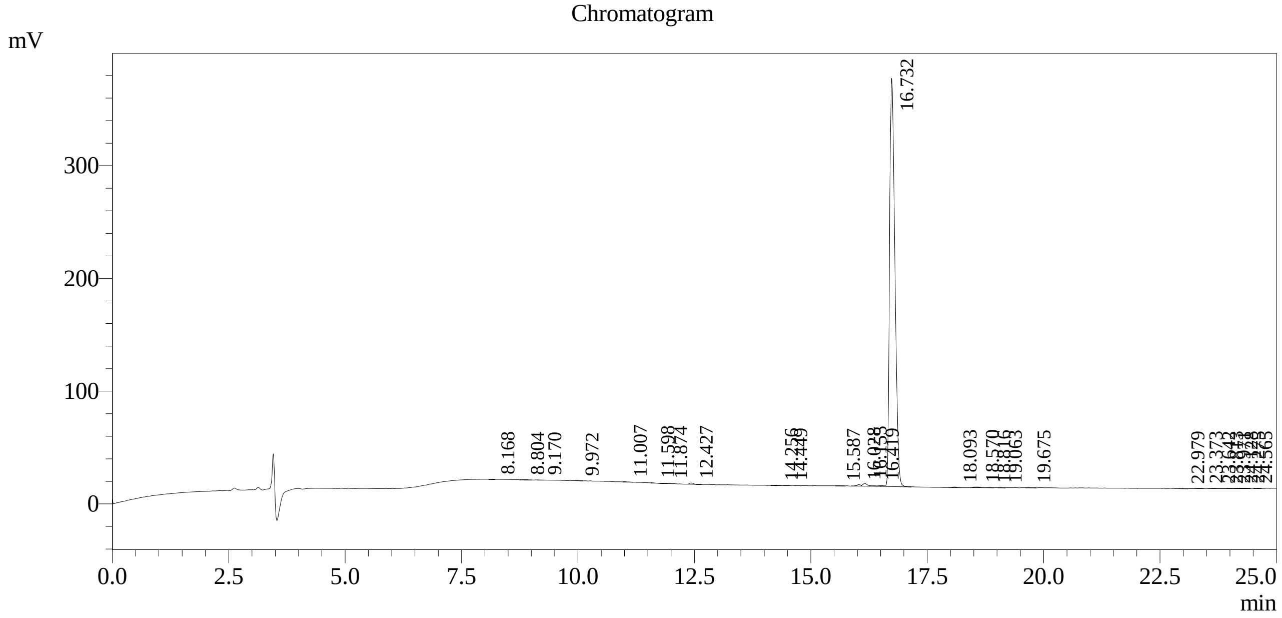

1 Det.A Ch1 / 220nm

Peak Table

Detector A Ch1 220nm

Peak#	Ret. Time	Area	Height	Area %
1	8.168	1025	192	0.034
2	8.804	1075	184	0.036
3	9.170	1002	206	0.034
4	9.972	1518	265	0.051
5	11.007	1043	178	0.035
6	11.598	1644	161	0.055
7	11.874	1174	161	0.040
8	12.427	6720	1106	0.226
9	14.256	1769	268	0.060
10	14.449	1581	210	0.053
11	15.587	1088	173	0.037
12	16.028	7196	1261	0.242
13	16.155	15942	2425	0.536
14	16.419	10762	920	0.362
15	16.732	2887501	362031	97.148
16	18.093	3944	597	0.133
17	18.570	6928	648	0.233
18	18.816	1945	251	0.065
19	19.063	1191	220	0.040
20	19.675	1283	207	0.043
21	22.979	1145	180	0.039
22	23.373	3368	298	0.113
23	23.642	2911	298	0.098
24	23.813	1171	191	0.039

Peak#	Ret. Time	Area	Height	Area %
25	23.971	2106	276	0.071
26	24.128	1508	250	0.051
27	24.275	2477	296	0.083
28	24.565	1249	263	0.042
Total		2972266	373717	100.000

Mass Spectrum

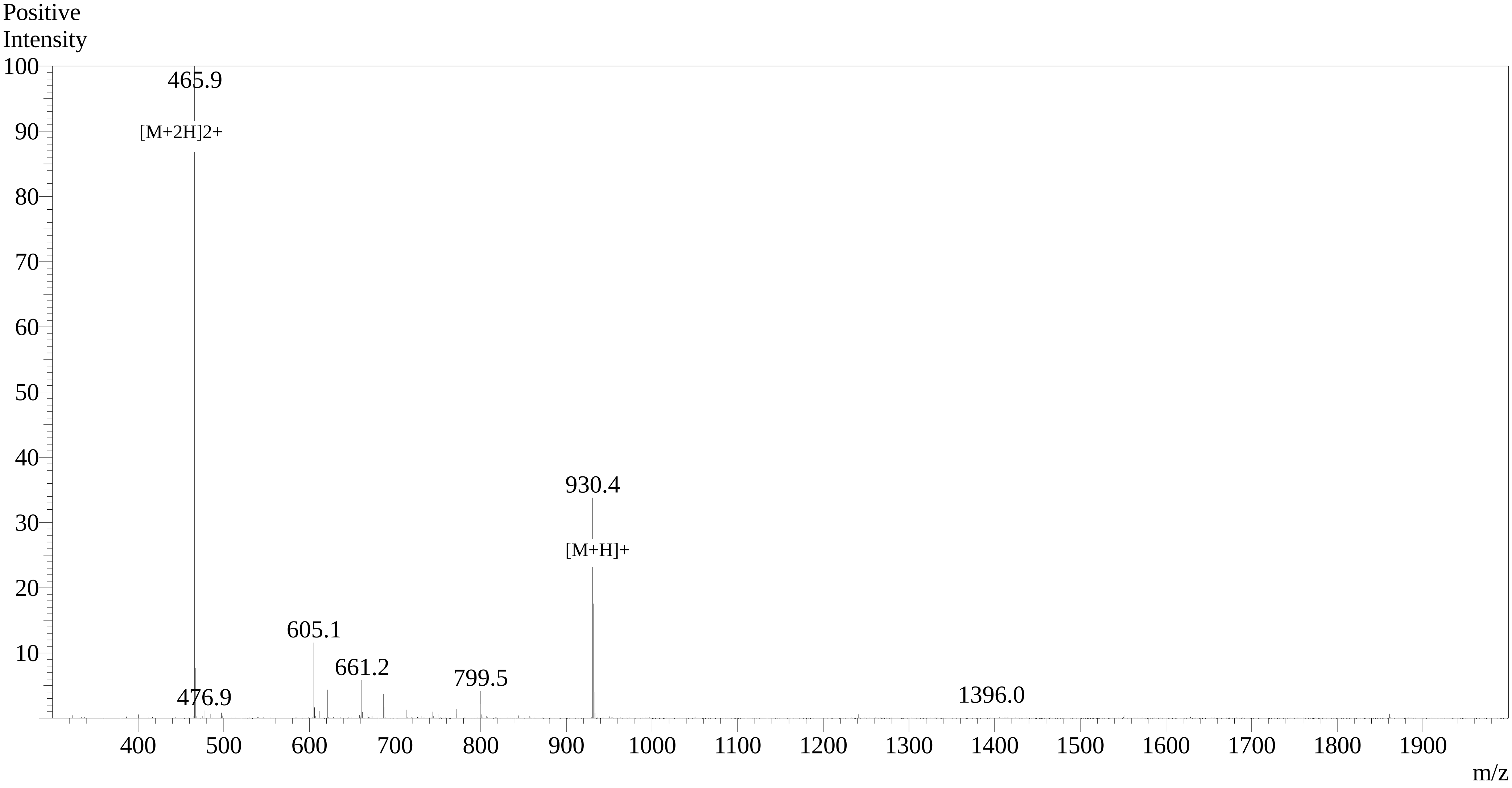

Sample Information	
Acquired by :	Gary
Month-Day Processed :	08/24/19
Time Processed :	22:28:11
Injection Volume :	0.3
Sample Name :	TBEV_NS5_Int_PBM3
Sample ID :	U877LEH160-7
Theoretical MW :	930.11
Observed MW :	929.8

Interface	:ESI
Nebulizing Gas Flow	:1.5L/min
CDL Temp	:250
Block Temp	:200

Equipment	: GK11010007
Interface Bias	: +4.5 kV
Drying Gas Flow	:5 L/min
T.Flow	:0.2 ml/min
B.conc	:50%H2O/50%MeOH

CERTIFICATE OF ANALYSIS

**Product Name**	NS5 internal (PBM4)
**Order ID**	U015HGD140_1
**Lot No.**	U015HGD140-1/PE0912
**Sequence**	EMYYSTAVTGNI
**Modification**	N/A
**Length**	12AA
**Storage**	-20°C
**Recommended Solvent***	ultrapure water
**comments**	TFA salt

**Test Items**	**Specifications**	**Results**
**Molecular Weight**	Theoretical MW: 1348.48	Consistent
**HPLC purity**	≥95.0%	96.0%
**Appearance**	White lyophilized powder	Conforms
**Gross Weight**	14 mg	14.0mg

*Note: Above recommended solvents for reference only. If there is any request for detailed dissolution conditions,we suggest you choose our 'Peptide Solubility Test Service'.

Caution:
For laboratory or further manufacturing use only. Not intended for household use. If you have any questions about the Certificate of Analysis, please contact our customer service representative at 1-877-436-7274 (Toll-Free), or 1-732-885-9188.

Certified by: Gary Chen
Peptide Production Director

Date:05-07-2021

Thank you for your patronage to our Peptide services! To maintain this working relationship, we shall be grateful if you can add our webpage URL into your lab website. As a token of appreciation, you will be rewarded by 1,000 EZcoupon™ points. For more information, please contact us by e-mail at web@genscript.com

Sample Name : NS5 internal (PBM4)
Sample ID :U015HGD140-1
Time Processed: 23:34:26
Year-Month-Day: 2021/5/3

Pump A : 0.065% trifluoroacetic in 100% water (v/v)
Pump B : 0.05% trifluoroacetic in 100% acetonitrile (v/v)
Total Flow:1 ml/min
<<Detector A>>
Wavelength Ch1 : 220 nm

<<LC Time Program>>

Time	Module	Command	Value	Comment
0.01	Pumps	Solvent B Conc.	5	
25.00	Pumps	Solvent B Conc.	65	
25.01	Pumps	Solvent B Conc.	95	
27.00	Pumps	Solvent B Conc.	95	
27.01	Pumps	Solvent B Conc.	5	
33.00	Pumps	Solvent B Conc.	5	
33.01	Controller	Stop		

<<Column Performance>>
<Detector A>
Column : Inertsil ODS-3 4.6 x 250 mm
Equipment:ZJ19010015

<Chromatogram>

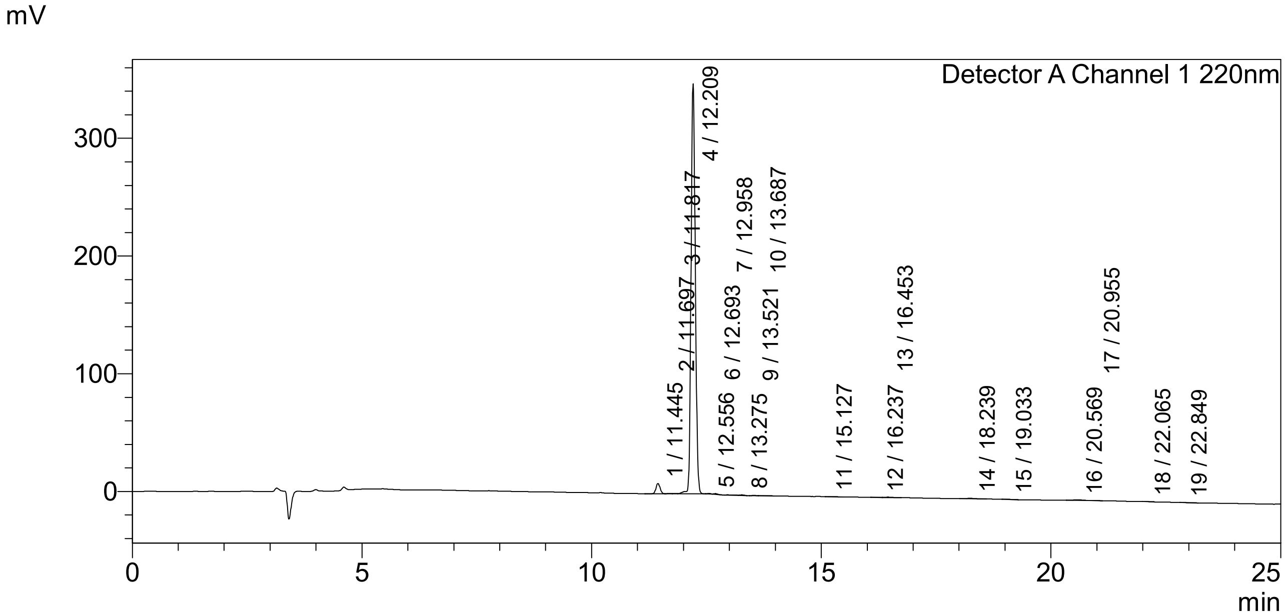

<Peak Table>

Detector A Channel 1 220nm

Peak#	Ret. Time	Area	Height	Area%
1	11.445	55820	8739	2.387
2	11.697	1666	235	0.071
3	11.817	1425	257	0.061
4	12.209	2244120	348352	95.976
5	12.556	1962	457	0.084
6	12.693	2741	566	0.117
7	12.958	1948	164	0.083
8	13.275	2503	449	0.107
9	13.521	1598	194	0.068
10	13.687	1874	164	0.080
11	15.127	1151	62	0.049
12	16.237	1384	132	0.059
13	16.453	3959	371	0.169
14	18.239	4283	324	0.183

Peak#	Ret. Time	Area	Height	Area%
15	19.033	1592	159	0.068
16	20.569	6633	415	0.284
17	20.955	1160	167	0.050
18	22.065	1354	187	0.058
19	22.849	1035	61	0.044
Total		2338208	361454	100.000

Mass Spectrum

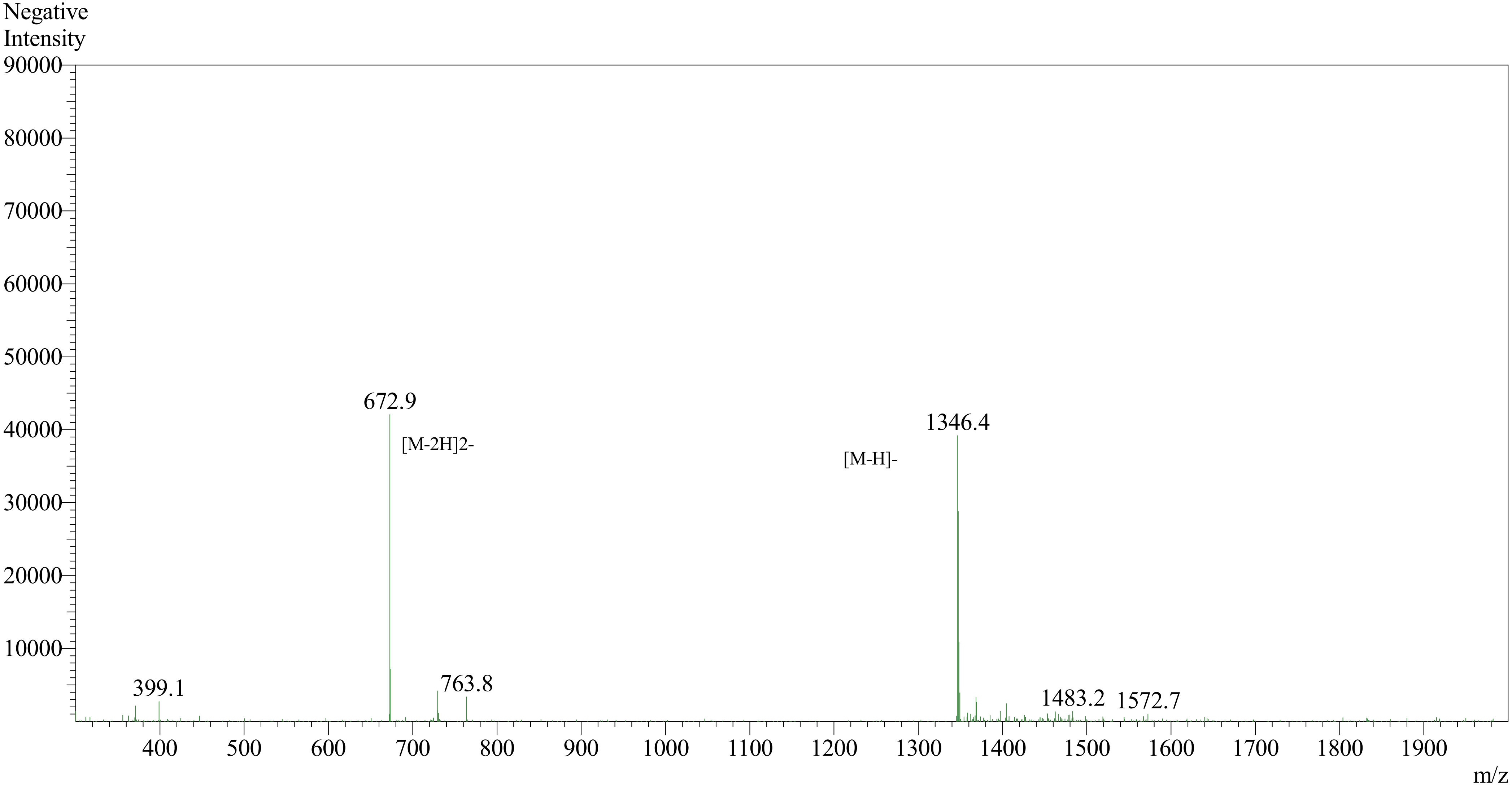

Sample Information
Month-Day Processed : 05/03/21
Time Processed : 21:33:45
Injection Volume : 0.5
Sample Name : NS5 internal (PBM4)
Sample ID : U015HGD140-1
Theoretical MW : 1348.48
Observed MW : 1347.4

Interface :ESI
Nebulizing Gas Flow :1.5L/min
CDL Temp :250
Block Temp :200

Equipment : GK11010007
Interface Bias : -3.5 kV
Drying Gas Flow :5 L/min
T.Flow :0.2 ml/min
B.conc :50%H2O/50%MeOH

Full wwPDB X-ray Structure Validation Report (i)

Feb 2, 2022 – 02:15 pm GMT

PDB ID	:	7QSA
Title	:	Structural basis on the interaction of Scribble PDZ domains with the Tick Born encephalitis virus (TBEV) NS5 protein
Deposited on	:	2022-01-13
Resolution	:	2.02 Å(reported)

This wwPDB validation report is for manuscript review

This is a Full wwPDB X-ray Structure Validation Report.

This report is produced by the wwPDB biocuration pipeline after annotation of the structure.

We welcome your comments at *validation@mail.wwpdb.org*
A user guide is available at
https://www.wwpdb.org/validation/2017/XrayValidationReportHelp
with specific help available everywhere you see the (i) symbol.

The following versions of software and data (see references (i)) were used in the production of this report:

MolProbity	:	4.02b-467
Xtriage (Phenix)	:	1.13
EDS	:	2.26
Percentile statistics	:	20191225.v01 (using entries in the PDB archive December 25th 2019)
Refmac	:	5.8.0267
CCP4	:	7.1.010 (Gargrove)
Ideal geometry (proteins)	:	Engh & Huber (2001)
Ideal geometry (DNA, RNA)	:	Parkinson et al. (1996)
Validation Pipeline (wwPDB-VP)	:	2.26

For Manuscript Review

1 Overall quality at a glance (i)

The following experimental techniques were used to determine the structure:
X-RAY DIFFRACTION

The reported resolution of this entry is 2.02 Å.

Percentile scores (ranging between 0-100) for global validation metrics of the entry are shown in the following graphic. The table shows the number of entries on which the scores are based.

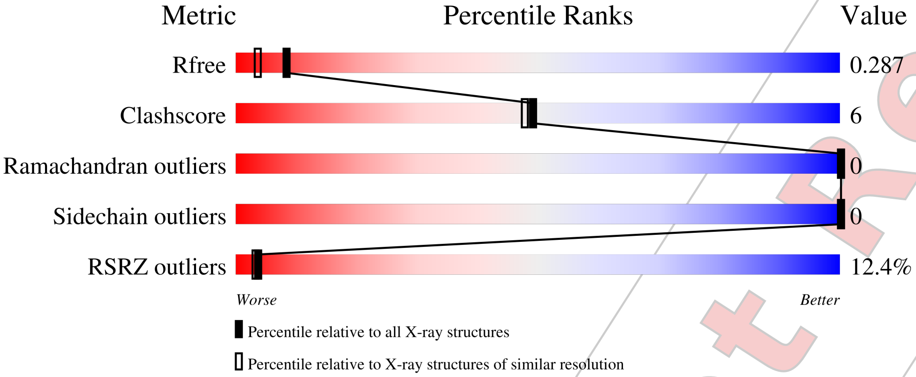

Metric	Whole archive (#Entries)	Similar resolution (#Entries, resolution range(Å))
R_{free}	130704	10434 (2.04-2.00)
Clashscore	141614	11643 (2.04-2.00)
Ramachandran outliers	138981	11493 (2.04-2.00)
Sidechain outliers	138945	11492 (2.04-2.00)
RSRZ outliers	127900	10220 (2.04-2.00)

The table below summarises the geometric issues observed across the polymeric chains and their fit to the electron density. The red, orange, yellow and green segments of the lower bar indicate the fraction of residues that contain outliers for >=3, 2, 1 and 0 types of geometric quality criteria respectively. A grey segment represents the fraction of residues that are not modelled. The numeric value for each fraction is indicated below the corresponding segment, with a dot representing fractions <=5% The upper red bar (where present) indicates the fraction of residues that have poor fit to the electron density. The numeric value is given above the bar.

Mol	Chain	Length	Quality of chain
1	A	92	10% / 84% 9% 8%
1	B	92	13% / 74% 11% 15%
2	C	8	62% 12% 25%

For Manuscript Review

2 Entry composition (i)

There are 3 unique types of molecules in this entry. The entry contains 2592 atoms, of which 1279 are hydrogens and 0 are deuteriums.

In the tables below, the ZeroOcc column contains the number of atoms modelled with zero occupancy, the AltConf column contains the number of residues with at least one atom in alternate conformation and the Trace column contains the number of residues modelled with at most 2 atoms.

- Molecule 1 is a protein called Protein scribble homolog.

Mol	Chain	Residues	Atoms					ZeroOcc	AltConf	Trace
			Total	C	H	N	O			
1	A	85	1309	401	666	125	117	0	0	0
1	B	78	1129	355	565	104	105	0	0	0

There are 2 discrepancies between the modelled and reference sequences:

Chain	Residue	Modelled	Actual	Comment	Reference
A	11	SER	-	expression tag	UNP Q14160
B	11	SER	-	expression tag	UNP Q14160

- Molecule 2 is a protein called RNA-directed RNA polymerase NS5.

Mol	Chain	Residues	Atoms					ZeroOcc	AltConf	Trace
			Total	C	H	N	O			
2	C	6	94	29	48	6	11	0	0	0

- Molecule 3 is water.

Mol	Chain	Residues	Atoms		ZeroOcc	AltConf
			Total	O		
3	A	38	38	38	0	0
3	B	20	20	20	0	0
3	C	2	2	2	0	0

For Manuscript Review

3 Residue-property plots (i)

These plots are drawn for all protein, RNA, DNA and oligosaccharide chains in the entry. The first graphic for a chain summarises the proportions of the various outlier classes displayed in the second graphic. The second graphic shows the sequence view annotated by issues in geometry and electron density. Residues are color-coded according to the number of geometric quality criteria for which they contain at least one outlier: green = 0, yellow = 1, orange = 2 and red = 3 or more. A red dot above a residue indicates a poor fit to the electron density (RSRZ > 2). Stretches of 2 or more consecutive residues without any outlier are shown as a green connector. Residues present in the sample, but not in the model, are shown in grey.

- Molecule 1: Protein scribble homolog

Chain A:

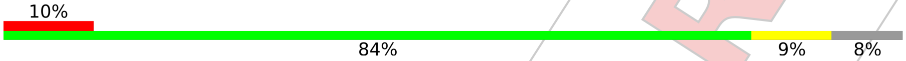

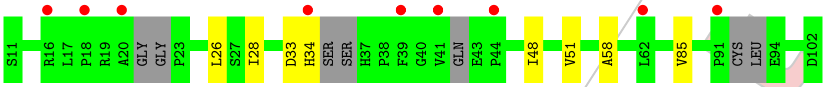

- Molecule 1: Protein scribble homolog

Chain B:

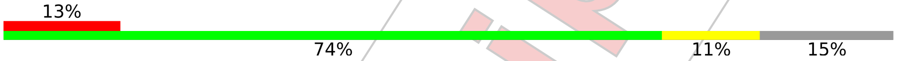

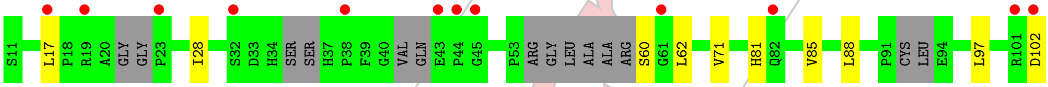

- Molecule 2: RNA-directed RNA polymerase NS5

Chain C:

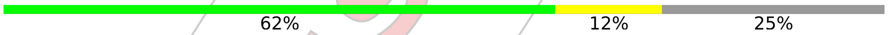

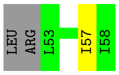

For Manuscript Review

4 Data and refinement statistics (i)

Property	Value	Source
Space group	H 3 2	Depositor
Cell constants<br>a, b, c, α, β, γ	69.95Å 69.95Å 201.61Å<br>90.00° 90.00° 120.00°	Depositor
Resolution (Å)	31.02 – 2.02<br>34.97 – 2.02	Depositor<br>EDS
% Data completeness<br>(in resolution range)	99.7 (31.02-2.02)<br>99.7 (34.97-2.02)	Depositor<br>EDS
R_{merge}	0.11	Depositor
R_{sym}	(Not available)	Depositor
$< I/\sigma(I) >$ [1]	0.95 (at 2.01Å)	Xtriage
Refinement program	PHENIX 1.20_4459	Depositor
R, R_{free}	0.253 , 0.287<br>0.253 , 0.287	Depositor<br>DCC
R_{free} test set	695 reflections (5.43%)	wwPDB-VP
Wilson B-factor (Å^2)	42.6	Xtriage
Anisotropy	0.101	Xtriage
Bulk solvent k_{sol}(e/Å^3), B_{sol}(Å^2)	(Not available) , (Not available)	EDS
L-test for twinning[2]	$< \|L\| > = 0.49$, $< L^2 > = 0.33$	Xtriage
Estimated twinning fraction	No twinning to report.	Xtriage
F_o,F_c correlation	0.93	EDS
Total number of atoms	2592	wwPDB-VP
Average B, all atoms (Å^2)	52.0	wwPDB-VP

Xtriage's analysis on translational NCS is as follows: *The largest off-origin peak in the Patterson function is 6.45% of the height of the origin peak. No significant pseudotranslation is detected.*

[1] Intensities estimated from amplitudes.

[2] Theoretical values of $< |L| >$, $< L^2 >$ for acentric reflections are 0.5, 0.333 respectively for untwinned datasets, and 0.375, 0.2 for perfectly twinned datasets.

For Manuscript Review

5 Model quality (i)

5.1 Standard geometry (i)

The Z score for a bond length (or angle) is the number of standard deviations the observed value is removed from the expected value. A bond length (or angle) with $|Z| > 5$ is considered an outlier worth inspection. RMSZ is the root-mean-square of all Z scores of the bond lengths (or angles).

Mol	Chain	Bond lengths		Bond angles	
		RMSZ	#|Z| >5	RMSZ	#|Z| >5
1	A	0.60	0/649	0.86	0/870
1	B	0.57	0/567	0.75	0/762
2	C	0.66	0/45	0.70	0/58
All	All	0.59	0/1261	0.81	0/1690

There are no bond length outliers.

There are no bond angle outliers.

There are no chirality outliers.

There are no planarity outliers.

5.2 Too-close contacts (i)

In the following table, the Non-H and H(model) columns list the number of non-hydrogen atoms and hydrogen atoms in the chain respectively. The H(added) column lists the number of hydrogen atoms added and optimized by MolProbity. The Clashes column lists the number of clashes within the asymmetric unit, whereas Symm-Clashes lists symmetry-related clashes.

Mol	Chain	Non-H	H(model)	H(added)	Clashes	Symm-Clashes
1	A	643	666	662	8	0
1	B	564	565	561	6	0
2	C	46	48	48	1	0
3	A	38	0	0	0	0
3	B	20	0	0	0	0
3	C	2	0	0	0	0
All	All	1313	1279	1271	15	0

The all-atom clashscore is defined as the number of clashes found per 1000 atoms (including hydrogen atoms). The all-atom clashscore for this structure is 6.

All (15) close contacts within the same asymmetric unit are listed below, sorted by their clash magnitude.

For Manuscript Review

Atom-1	Atom-2	Interatomic distance (Å)	Clash overlap (Å)
1:A:28:ILE:HD11	1:A:85:VAL:HG22	1.85	0.59
1:A:28:ILE:HD11	1:A:85:VAL:CG2	2.40	0.51
1:A:28:ILE:HG22	1:A:48:ILE:HD13	1.92	0.50
1:A:33:ASP:O	1:A:34:HIS:HB2	2.11	0.50
1:B:81:HIS:O	1:B:85:VAL:HG23	2.12	0.49
1:B:71:VAL:HG22	1:B:97:LEU:CD2	2.44	0.48
2:C:57:ILE:HD12	2:C:57:ILE:N	2.29	0.48
1:A:26:LEU:CD1	1:A:28:ILE:HG23	2.44	0.47
1:A:26:LEU:HD12	1:A:26:LEU:C	2.35	0.47
1:B:102:ASP:N	1:B:102:ASP:OD1	2.50	0.44
1:B:62:LEU:HD11	1:B:97:LEU:HD12	2.01	0.43
1:B:17:LEU:HD21	1:B:60:SER:O	2.19	0.42
1:A:51:VAL:CG1	1:A:58:ALA:HB2	2.49	0.42
1:B:28:ILE:HD13	1:B:88:LEU:HD12	2.02	0.42
1:A:51:VAL:HG12	1:A:58:ALA:HB2	2.02	0.41

There are no symmetry-related clashes.

5.3 Torsion angles (i)

5.3.1 Protein backbone (i)

In the following table, the Percentiles column shows the percent Ramachandran outliers of the chain as a percentile score with respect to all X-ray entries followed by that with respect to entries of similar resolution.

The Analysed column shows the number of residues for which the backbone conformation was analysed, and the total number of residues.

Mol	Chain	Analysed	Favoured	Allowed	Outliers	Percentiles	
1	A	75/92 (82%)	74 (99%)	1 (1%)	0	100	100
1	B	66/92 (72%)	65 (98%)	1 (2%)	0	100	100
2	C	4/8 (50%)	4 (100%)	0	0	100	100
All	All	145/192 (76%)	143 (99%)	2 (1%)	0	100	100

There are no Ramachandran outliers to report.

For Manuscript Review

5.3.2 Protein sidechains (i)

In the following table, the Percentiles column shows the percent sidechain outliers of the chain as a percentile score with respect to all X-ray entries followed by that with respect to entries of similar resolution.

The Analysed column shows the number of residues for which the sidechain conformation was analysed, and the total number of residues.

Mol	Chain	Analysed	Rotameric	Outliers	Percentiles	
1	A	69/74 (93%)	69 (100%)	0	100	100
1	B	59/74 (80%)	59 (100%)	0	100	100
2	C	6/8 (75%)	6 (100%)	0	100	100
All	All	134/156 (86%)	134 (100%)	0	100	100

There are no protein residues with a non-rotameric sidechain to report.

Sometimes sidechains can be flipped to improve hydrogen bonding and reduce clashes. There are no such sidechains identified.

5.3.3 RNA (i)

There are no RNA molecules in this entry.

5.4 Non-standard residues in protein, DNA, RNA chains (i)

There are no non-standard protein/DNA/RNA residues in this entry.

5.5 Carbohydrates (i)

There are no monosaccharides in this entry.

5.6 Ligand geometry (i)

There are no ligands in this entry.

5.7 Other polymers (i)

There are no such residues in this entry.

For Manuscript Review

5.8 Polymer linkage issues (i)

There are no chain breaks in this entry.

For Manuscript Review

6 Fit of model and data (i)

6.1 Protein, DNA and RNA chains (i)

In the following table, the column labelled '#RSRZ> 2' contains the number (and percentage) of RSRZ outliers, followed by percent RSRZ outliers for the chain as percentile scores relative to all X-ray entries and entries of similar resolution. The OWAB column contains the minimum, median, 95th percentile and maximum values of the occupancy-weighted average B-factor per residue. The column labelled 'Q< 0.9' lists the number of (and percentage) of residues with an average occupancy less than 0.9.

Mol	Chain	Analysed	<RSRZ>	#RSRZ>2			OWAB($Å^2$)	Q<0.9
1	A	85/92 (92%)	0.81	9 (10%)	6	5	33, 41, 59, 74	0
1	B	78/92 (84%)	1.05	12 (15%)	2	1	37, 51, 70, 153	0
2	C	6/8 (75%)	0.39	0	100	100	36, 40, 45, 60	0
All	All	169/192 (88%)	0.91	21 (12%)	4	3	33, 44, 68, 153	0

All (21) RSRZ outliers are listed below:

Mol	Chain	Res	Type	RSRZ
1	B	23	PRO	7.9
1	A	34	HIS	4.8
1	B	44	PRO	4.1
1	B	43	GLU	3.6
1	B	17	LEU	3.6
1	A	41	VAL	3.5
1	A	62	LEU	3.1
1	B	82	GLN	2.9
1	B	19	ARG	2.8
1	A	20	ALA	2.8
1	B	102	ASP	2.5
1	B	38	PRO	2.5
1	A	44	PRO	2.4
1	A	16	ARG	2.4
1	B	45	GLY	2.3
1	B	101	ARG	2.3
1	A	91	PRO	2.2
1	B	61	GLY	2.2
1	A	39	PHE	2.2
1	B	32	SER	2.0
1	A	18	PRO	2.0

For Manuscript Review

6.2 Non-standard residues in protein, DNA, RNA chains (i)

There are no non-standard protein/DNA/RNA residues in this entry.

6.3 Carbohydrates (i)

There are no monosaccharides in this entry.

6.4 Ligands (i)

There are no ligands in this entry.

6.5 Other polymers (i)

There are no such residues in this entry.

For Manuscript Review

Full wwPDB X-ray Structure Validation Report (i)

Jan 14, 2022 – 12:07 pm GMT

PDB ID	:	7QSB
Title	:	Structural basis on the interaction of Scribble PDZ domains with the Tick Born encephalitis virus (TBEV) NS5 protein
Deposited on	:	2022-01-13
Resolution	:	1.84 Å(reported)

This is a Full wwPDB X-ray Structure Validation Report.

This report is produced by the wwPDB biocuration pipeline after annotation of the structure.

We welcome your comments at *validation@mail.wwpdb.org*
A user guide is available at
https://www.wwpdb.org/validation/2017/XrayValidationReportHelp
with specific help available everywhere you see the (i) symbol.

The following versions of software and data (see references (i)) were used in the production of this report:

MolProbity	:	4.02b-467
Xtriage (Phenix)	:	1.13
EDS	:	2.24
Percentile statistics	:	20191225.v01 (using entries in the PDB archive December 25th 2019)
Refmac	:	5.8.0267
CCP4	:	7.1.010 (Gargrove)
Ideal geometry (proteins)	:	Engh & Huber (2001)
Ideal geometry (DNA, RNA)	:	Parkinson et al. (1996)
Validation Pipeline (wwPDB-VP)	:	2.24

CONFIDENTIAL VALIDATION REPORT

1 Overall quality at a glance (i)

The following experimental techniques were used to determine the structure:
X-RAY DIFFRACTION

The reported resolution of this entry is 1.84 Å.

Percentile scores (ranging between 0-100) for global validation metrics of the entry are shown in the following graphic. The table shows the number of entries on which the scores are based.

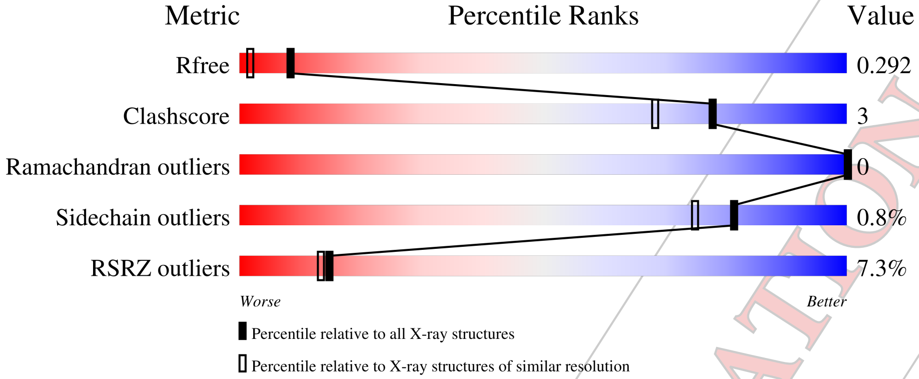

Metric	Whole archive (#Entries)	Similar resolution (#Entries, resolution range(Å))
R_{free}	130704	4003 (1.86-1.82)
Clashscore	141614	4233 (1.86-1.82)
Ramachandran outliers	138981	4185 (1.86-1.82)
Sidechain outliers	138945	4186 (1.86-1.82)
RSRZ outliers	127900	3957 (1.86-1.82)

The table below summarises the geometric issues observed across the polymeric chains and their fit to the electron density. The red, orange, yellow and green segments of the lower bar indicate the fraction of residues that contain outliers for >=3, 2, 1 and 0 types of geometric quality criteria respectively. A grey segment represents the fraction of residues that are not modelled. The numeric value for each fraction is indicated below the corresponding segment, with a dot representing fractions <=5% The upper red bar (where present) indicates the fraction of residues that have poor fit to the electron density. The numeric value is given above the bar.

Mol	Chain	Length	Quality of chain
1	A	92	5% / 87% • 9%
1	B	92	8% / 77% 10% 13%

CONFIDENTIAL VALIDATION REPORT

2 Entry composition (i)

There are 2 unique types of molecules in this entry. The entry contains 2485 atoms, of which 1226 are hydrogens and 0 are deuteriums.

In the tables below, the ZeroOcc column contains the number of atoms modelled with zero occupancy, the AltConf column contains the number of residues with at least one atom in alternate conformation and the Trace column contains the number of residues modelled with at most 2 atoms.

- Molecule 1 is a protein called Protein scribble homolog.

Mol	Chain	Residues	Atoms						ZeroOcc	AltConf	Trace
			Total	C	H	N	O	S			
1	A	84	1266	386	645	121	113	1	0	0	0
1	B	80	1157	361	581	107	107	1	0	0	0

There are 2 discrepancies between the modelled and reference sequences:

Chain	Residue	Modelled	Actual	Comment	Reference
A	11	SER	-	expression tag	UNP Q14160
B	11	SER	-	expression tag	UNP Q14160

- Molecule 2 is water.

Mol	Chain	Residues	Atoms		ZeroOcc	AltConf
			Total	O		
2	A	38	38	38	0	0
2	B	24	24	24	0	0

CONFIDENTIAL VALIDATION REPORT

3 Residue-property plots (i)

These plots are drawn for all protein, RNA, DNA and oligosaccharide chains in the entry. The first graphic for a chain summarises the proportions of the various outlier classes displayed in the second graphic. The second graphic shows the sequence view annotated by issues in geometry and electron density. Residues are color-coded according to the number of geometric quality criteria for which they contain at least one outlier: green = 0, yellow = 1, orange = 2 and red = 3 or more. A red dot above a residue indicates a poor fit to the electron density (RSRZ > 2). Stretches of 2 or more consecutive residues without any outlier are shown as a green connector. Residues present in the sample, but not in the model, are shown in grey.

- Molecule 1: Protein scribble homolog

Chain A: 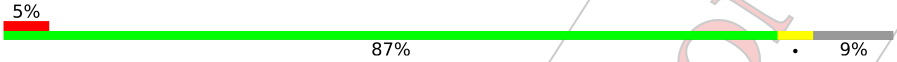

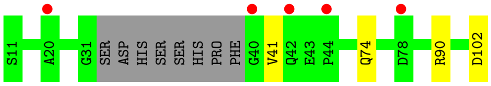

- Molecule 1: Protein scribble homolog

Chain B: 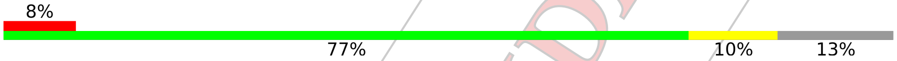

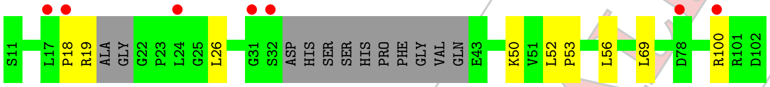

CONFIDENTIAL VALIDATION REPORT

4 Data and refinement statistics (i)

Property	Value	Source
Space group	P 21 21 21	Depositor
Cell constants<br>a, b, c, α, β, γ	36.88Å 60.88Å 64.90Å<br>90.00° 90.00° 90.00°	Depositor
Resolution (Å)	31.55 – 1.84<br>31.55 – 1.84	Depositor<br>EDS
% Data completeness<br>(in resolution range)	98.0 (31.55-1.84)<br>98.0 (31.55-1.84)	Depositor<br>EDS
R_{merge}	0.03	Depositor
R_{sym}	(Not available)	Depositor
$< I/\sigma(I) >$ [1]	1.18 (at 1.84Å)	Xtriage
Refinement program	PHENIX 1.15.2_3472	Depositor
R, R_{free}	0.248 , 0.292<br>0.247 , 0.292	Depositor<br>DCC
R_{free} test set	644 reflections (5.01%)	wwPDB-VP
Wilson B-factor (Å^2)	27.5	Xtriage
Anisotropy	0.600	Xtriage
Bulk solvent k_{sol}(e/Å^3), B_{sol}(Å^2)	(Not available) , (Not available)	EDS
L-test for twinning[2]	$< \|L\| > = 0.51$, $< L^2 > = 0.35$	Xtriage
Estimated twinning fraction	No twinning to report.	Xtriage
F_o,F_c correlation	0.94	EDS
Total number of atoms	2485	wwPDB-VP
Average B, all atoms (Å^2)	37.0	wwPDB-VP

Xtriage's analysis on translational NCS is as follows: *The analyses of the Patterson function reveals a significant off-origin peak that is 42.21 % of the origin peak, indicating pseudo-translational symmetry. The chance of finding a peak of this or larger height randomly in a structure without pseudo-translational symmetry is equal to 2.1087e-04. The detected translational NCS is most likely also responsible for the elevated intensity ratio.*

[1] Intensities estimated from amplitudes.
[2] Theoretical values of $< |L| >$, $< L^2 >$ for acentric reflections are 0.5, 0.333 respectively for untwinned datasets, and 0.375, 0.2 for perfectly twinned datasets.

CONFIDENTIAL VALIDATION REPORT

5 Model quality (i)

5.1 Standard geometry (i)

The Z score for a bond length (or angle) is the number of standard deviations the observed value is removed from the expected value. A bond length (or angle) with $|Z| > 5$ is considered an outlier worth inspection. RMSZ is the root-mean-square of all Z scores of the bond lengths (or angles).

Mol	Chain	Bond lengths		Bond angles	
		RMSZ	#$\|Z\|$ >5	RMSZ	#$\|Z\|$ >5
1	A	0.30	0/626	0.48	0/844
1	B	0.29	0/580	0.49	0/784
All	All	0.30	0/1206	0.49	0/1628

There are no bond length outliers.

There are no bond angle outliers.

There are no chirality outliers.

There are no planarity outliers.

5.2 Too-close contacts (i)

In the following table, the Non-H and H(model) columns list the number of non-hydrogen atoms and hydrogen atoms in the chain respectively. The H(added) column lists the number of hydrogen atoms added and optimized by MolProbity. The Clashes column lists the number of clashes within the asymmetric unit, whereas Symm-Clashes lists symmetry-related clashes.

Mol	Chain	Non-H	H(model)	H(added)	Clashes	Symm-Clashes
1	A	621	645	655	2	0
1	B	576	581	594	6	0
2	A	38	0	0	0	0
2	B	24	0	0	0	0
All	All	1259	1226	1249	7	0

The all-atom clashscore is defined as the number of clashes found per 1000 atoms (including hydrogen atoms). The all-atom clashscore for this structure is 3.

All (7) close contacts within the same asymmetric unit are listed below, sorted by their clash magnitude.

Atom-1	Atom-2	Interatomic distance (Å)	Clash overlap (Å)
1:B:18:PRO:HD2	1:B:56:LEU:HD11	1.77	0.65

Continued on next page...

CONFIDENTIAL VALIDATION REPORT

Continued from previous page...

Atom-1	Atom-2	Interatomic distance (Å)	Clash overlap (Å)
1:B:26:LEU:HD12	1:B:26:LEU:C	2.31	0.52
1:B:52:LEU:HD12	1:B:53:PRO:HD2	1.97	0.47
1:B:18:PRO:O	1:B:19:ARG:C	2.55	0.46
1:B:69:LEU:HD11	1:B:100:ARG:HB2	1.98	0.45
1:A:74:GLN:OE1	1:A:90:ARG:NH1	2.51	0.44
1:A:102:ASP:OD2	1:B:50:LYS:HE3	2.21	0.41

There are no symmetry-related clashes.

5.3 Torsion angles (i)

5.3.1 Protein backbone (i)

In the following table, the Percentiles column shows the percent Ramachandran outliers of the chain as a percentile score with respect to all X-ray entries followed by that with respect to entries of similar resolution.

The Analysed column shows the number of residues for which the backbone conformation was analysed, and the total number of residues.

Mol	Chain	Analysed	Favoured	Allowed	Outliers	Percentiles	
1	A	80/92 (87%)	80 (100%)	0	0	100	100
1	B	74/92 (80%)	74 (100%)	0	0	100	100
All	All	154/184 (84%)	154 (100%)	0	0	100	100

There are no Ramachandran outliers to report.

5.3.2 Protein sidechains (i)

In the following table, the Percentiles column shows the percent sidechain outliers of the chain as a percentile score with respect to all X-ray entries followed by that with respect to entries of similar resolution.

The Analysed column shows the number of residues for which the sidechain conformation was analysed, and the total number of residues.

Mol	Chain	Analysed	Rotameric	Outliers	Percentiles	
1	A	66/74 (89%)	65 (98%)	1 (2%)	65	52
1	B	61/74 (82%)	61 (100%)	0	100	100

Continued on next page...

CONFIDENTIAL VALIDATION REPORT

Continued from previous page...

Mol	Chain	Analysed	Rotameric	Outliers	Percentiles	
All	All	127/148 (86%)	126 (99%)	1 (1%)	81	75

All (1) residues with a non-rotameric sidechain are listed below:

Mol	Chain	Res	Type
1	A	41	VAL

Sometimes sidechains can be flipped to improve hydrogen bonding and reduce clashes. There are no such sidechains identified.

5.3.3 RNA (i)

There are no RNA molecules in this entry.

5.4 Non-standard residues in protein, DNA, RNA chains (i)

There are no non-standard protein/DNA/RNA residues in this entry.

5.5 Carbohydrates (i)

There are no monosaccharides in this entry.

5.6 Ligand geometry (i)

There are no ligands in this entry.

5.7 Other polymers (i)

There are no such residues in this entry.

5.8 Polymer linkage issues (i)

There are no chain breaks in this entry.

CONFIDENTIAL VALIDATION REPORT

6 Fit of model and data (i)

6.1 Protein, DNA and RNA chains (i)

In the following table, the column labelled '#RSRZ> 2' contains the number (and percentage) of RSRZ outliers, followed by percent RSRZ outliers for the chain as percentile scores relative to all X-ray entries and entries of similar resolution. The OWAB column contains the minimum, median, 95th percentile and maximum values of the occupancy-weighted average B-factor per residue. The column labelled 'Q< 0.9' lists the number of (and percentage) of residues with an average occupancy less than 0.9.

Mol	Chain	Analysed	<RSRZ>	#RSRZ>2			OWAB(Å^2)	Q<0.9
1	A	84/92 (91%)	0.60	5 (5%)	21	19	22, 31, 48, 58	0
1	B	80/92 (86%)	0.65	7 (8%)	10	8	22, 34, 53, 82	0
All	All	164/184 (89%)	0.62	12 (7%)	15	13	22, 32, 51, 82	0

All (12) RSRZ outliers are listed below:

Mol	Chain	Res	Type	RSRZ
1	B	32	SER	4.9
1	B	18	PRO	4.7
1	A	40	GLY	3.6
1	B	17	LEU	3.3
1	B	78	ASP	3.2
1	A	78	ASP	3.0
1	A	20	ALA	2.7
1	B	24	LEU	2.5
1	A	42	GLN	2.4
1	B	31	GLY	2.2
1	A	44	PRO	2.1
1	B	100	ARG	2.0

6.2 Non-standard residues in protein, DNA, RNA chains (i)

There are no non-standard protein/DNA/RNA residues in this entry.

6.3 Carbohydrates (i)

There are no monosaccharides in this entry.

CONFIDENTIAL VALIDATION REPORT

6.4 Ligands (i)

There are no ligands in this entry.

6.5 Other polymers (i)

There are no such residues in this entry.

CONFIDENTIAL VALIDATION REPORT
